# Functional Connectivity in Multiple Sclerosis: Recent Findings and Future Directions

**DOI:** 10.3389/fneur.2018.00828

**Published:** 2018-10-11

**Authors:** Marlene Tahedl, Seth M. Levine, Mark W. Greenlee, Robert Weissert, Jens V. Schwarzbach

**Affiliations:** ^1^Department of Psychiatry and Psychotherapy, University of Regensburg, Regensburg, Germany; ^2^Institute for Experimental Psychology, University of Regensburg, Regensburg, Germany; ^3^Department of Neurology, University of Regensburg, Regensburg, Germany

**Keywords:** fMRI, functional connectivity, multiple sclerosis, resting state, neuroimaging biomarker

## Abstract

Multiple sclerosis is a debilitating disorder resulting from scattered lesions in the central nervous system. Because of the high variability of the lesion patterns between patients, it is difficult to relate existing biomarkers to symptoms and their progression. The scattered nature of lesions in multiple sclerosis offers itself to be studied through the lens of network analyses. Recent research into multiple sclerosis has taken such a network approach by making use of functional connectivity. In this review, we briefly introduce measures of functional connectivity and how to compute them. We then identify several common observations resulting from this approach: (a) high likelihood of altered connectivity in deep-gray matter regions, (b) decrease of brain modularity, (c) hemispheric asymmetries in connectivity alterations, and (d) correspondence of behavioral symptoms with task-related and task-unrelated networks. We propose incorporating such connectivity analyses into longitudinal studies in order to improve our understanding of the underlying mechanisms affected by multiple sclerosis, which can consequently offer a promising route to individualizing imaging-related biomarkers for multiple sclerosis.

## Introduction

### Multiple sclerosis

Multiple sclerosis (MS) is the most common non-traumatic neurological disorder among young adults (1). Traditionally, it is considered an autoimmune disease, which arises from an autoreactive response of an adaptive immune system against antigens derived from the central nervous system (CNS). Autoreactive lymphocytes transmigrate through the blood-brain barrier and subsequently induce lesions. Such lesions are characterized by an inflammatory process and a varying degree of demyelination and neuronal or axonal damage (2, 3), depending on the stage and severity of the immune attack and the tissue susceptibility.

Using the contrast-agent gadolinium (GD), which can cross the blood-brain barrier, acute inflammation can be visualized with magnetic resonance imaging (MRI), for an average of three weeks (4). A subacute and an inactive chronic phase often follow, wherein the lesion volume initially diminishes, possibly due to remyelination, and subsequently stabilizes. Using T2-FLAIR weighted MRI without contrast-agent is typical for monitoring the post-active phases, in which plaques appear as hyperintensities (5). During the course of the disease, a widespread lesion pattern evolves which differs for each patient (6). For the diagnosis of MS, two lesion-based criteria, which can be assessed by MRI, must be met: (a) lesions must be spread across different CNS regions (i.e., dissemination in space), and (b) new lesions must evolve over time, as compared to a baseline scan (i.e., dissemination in time). The appearance of only one GD-enhancing lesion in an otherwise lesion-free MRI scan is diagnostic of a condition often preceding clinically definite MS, called clinically isolated syndrome (CIS) (7).

Depending on the location of lesions within the CNS, a wide range of different neurological symptoms arises (8). However, the majority of lesions develops without presence of clinical symptoms (9). Acute bouts or relapses are defined as episodes of neurological impairment lasting at least 24 h, followed by varying degrees of remission (7). GD-enhanced MRI is used to identify bout/relapse-associated lesions. Relapsing-remitting MS (RRMS) is the most common disease course of MS, which often develops into a condition of continuously increasing disability, with or without superimposed relapses, after a range of about 10 to 15 years (secondary-progressive MS, SPMS). Less frequently, such increasing disability is observed from the diagnosis onwards (primary-progressive MS, PPMS). Currently the clinical course is described as active or inactive and progressive or non-progressive (10).

Often, a correct diagnosis of an individual's MS phenotype is only possible in retrospect. For example, the transition from relapsing to progressive MS is defined as continuously increasing disability with few or no superimposed (i.e., additional) relapses (10). The main diagnostic tools to assess disability in MS are the Expanded Disability Status Scale [EDSS (11)] and the Multiple Sclerosis Functional Composite [MSFC, (12)]. The EDSS rates performance in eight different functional systems, such as motor or sensory-related, while the MSFC rates ambulatory, manual, and cognitive performance. However, the assessment of such scales is rarely performed more than once a year, which limits the ability to pinpoint the onset of secondary progressive MS (10). Additionally, the distinction between onset-relapsing and onset-progressive subtypes requires longitudinal disease monitoring.

Another limitation in the prognosis of individual patients is how one accurately rates individual disease development, given that the pathological substrates and pace of disability accumulation are vastly heterogeneous even within subtypes [e.g., (13)]. Some of the currently used biomarkers in MS diagnosis/prognosis are obtained from either body fluids, such as neurofilament and oligoclonal bands (14), or MRI, such as T2 white matter lesion load (number × volume), the number and occurrence of GD-enhancing lesions, gray matter atrophy, or the presence of low-signal intensities on T1-weighted images, which is indicative of permanent tissue damage (15). However, the prognostic value of many of these biomarkers is of limited degree [see (16) on the clinico-radiological paradox], especially on an individual level (14, 15, 17, 18).

Furthermore, MRI-related biomarkers of MS suffer from the fact that different types of tissue damage occur at widespread locations in the CNS, with the highest incidence for lesions in the optic nerves, periventricular white matter, brainstem, cerebellum, and spinal cord white matter (19). Neurodegenerative and inflammatory processes in the gray matter are similarly prevalent in MS patients (3). However, lesions that affect specific white matter tracts (in particular those traversed by fibers involved in motor functions and near the corpus callosum) have been associated with a higher risk of clinical conversion of CIS to MS in the short term (20), while diffuse neurodegeneration, which does not appear to correlate with lesion load (19), has been linked to both physical and cognitive deficits in MS (20, 21). Nevertheless, attempts to relate such markers to diagnoses and prognoses of MS have been met with limited success, which is possibly due to the fact that these biomarkers are generally based on locationist/static information in a single MR image. As such, this review discusses connectivity based measures—a technique for furthering our understanding of the systems-level effects of MS—which we synthesize into four commonly observed motifs.

### Connectivity at a glance

The field of cognitive neuroscience has demonstrated that some cognitive and motor functions are localized in such a way that even a lesion in a single area can disrupt that function. For example a lesion in area V5/hMT+ of the visual cortex, which is associated with the perception of motion (22, 23) can lead to akinetopsia or motion blindness (24). However, other functions, such as mood, appear to be more distributed (i.e., they rely on a network of several brain regions), such that a lesion in a contributing area may lead to more subtle behavioral or cognitive defects. This problem is exacerbated by the fact that not only can a lesion have different effects on a function, but also multiple lesions can affect multiple cognitive functions and their network interactions. Additionally, a more generic, widespread breakdown (e.g., as a result of gray matter atrophy) of such a highly connected network resulting from atrophy processes may result in unpredictable effects on the system's underlying cognitive functions (25). The common hypothesis of the papers we review here is that MS changes how affected brain regions communicate with other brain areas: i.e., how their functional connectivity (FC) changes. Given this network-structure of the brain, it follows that analyses based on measures of connectivity (e.g., FC) would be promising for studying neural disorders. Thus, this insight motivates our review to focus on changes of brain connectivity in MS.

Connectivity is a generic term embracing all possible ways connections can emerge in a neural system (26). The neuroscientific study of connectivity distinguishes two types of connectivity: structural and FC. Structural connectivity (SC) of the brain is the connection strength between areas, e.g., measured with imaging methods such as diffusion tensor imaging (DTI). The scientific study of SC assumes that SC constrains brain-function and mental operations in a meaningful way. FC on the other hand, is the statistical association between time series of physiological signals, here the blood-oxygenation-level dependent (BOLD) response, from different parts of the brain. The concept of FC is based on the assumption that brain regions exhibiting a similar temporal evolution of activity share information and are thereby connected in a functional manner (27, 28).

### Calculating functional connectivity

**Figure 1:**
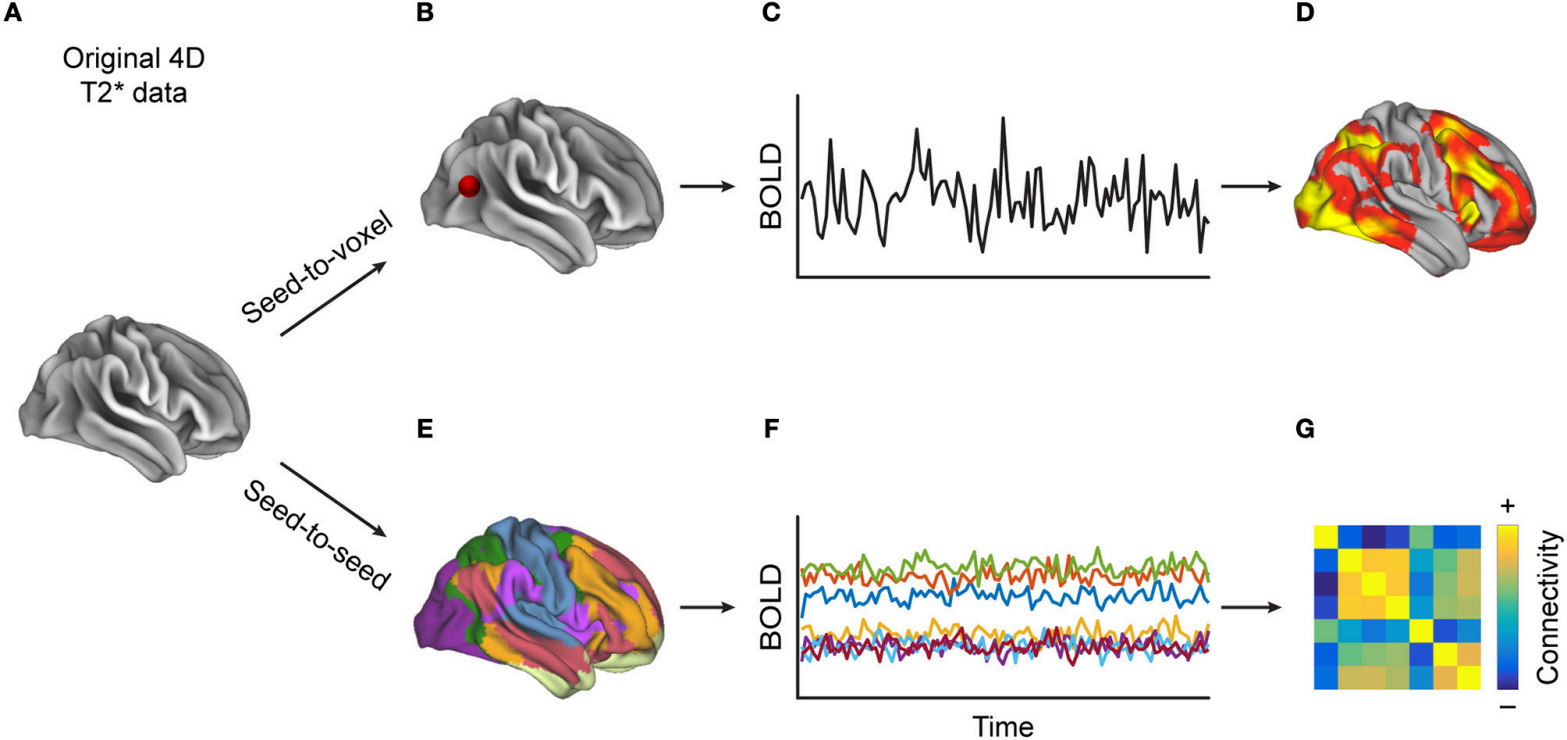
Methods for calculating correlation-based functional connectivity (FC). Starting with **(A)** a 4D-T2*-weighted dataset, when performing **(B)** a seed-to-voxel analysis, **(C)** the extracted timecourse (TC) from the seed region (depicted by the red dot) is correlated with the TCs from each of the other voxels, resulting in **(D)** a heat map, to which one can then apply a statistical threshold in order to reveal regions whose TCs are most similar to that of the seed region. Alternatively, **(E)** in a seed-to-seed-analysis, one parcels the brain into predefined atlas regions, extracts **(F)** the averaged TCs of those regions, and then computes the pairwise correlations between all regions' TCs, which can be visualized as **(G)** a FC matrix. One can also apply a statistical threshold to such a connectivity matrix.

**Figure 2:**
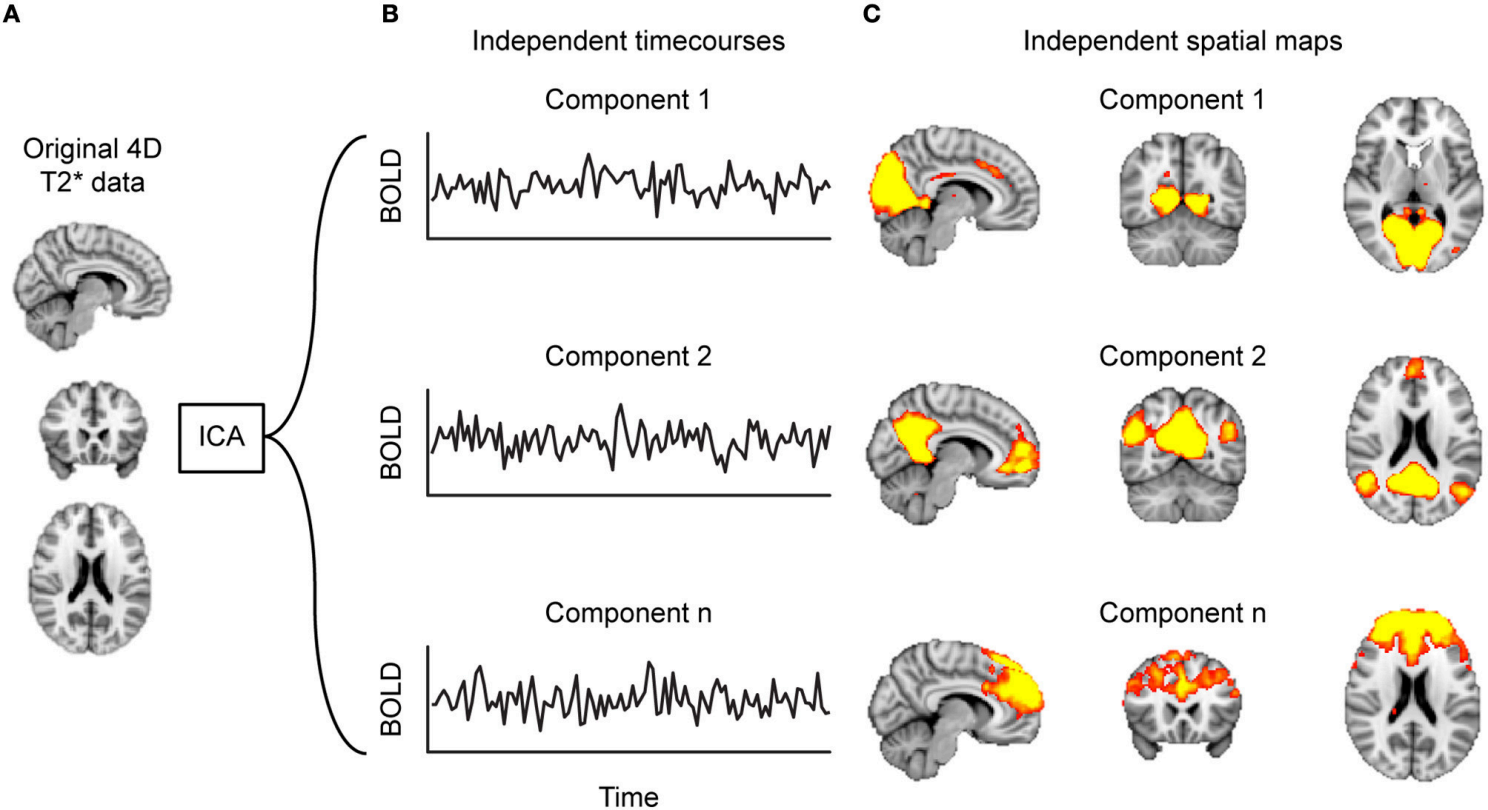
Decomposing functional connectivity (FC) with independent component analysis (ICA). Starting at **(A)** the 4D-T2*-weighted dataset, **(B)** the ICA algorithm identifies timecourses (TCs) that are spatially independent, referred to as components. These components can be represented as **(C)** heat maps projected on a brain. Such component maps can then form the basis of between-group analyses.

FC is a statistical concept that describes the association of timecourse data. The neuroimaging community has used several approaches to compute FC, such as correlation-analyses (Figure 1) between seed voxels and the rest of the brain (seed-to-voxel), between timecourses of all individual voxels (voxel-to-voxel) or between averaged timecourses of predefined regions of interest (seed-to-seed) or linear decomposition techniques of the entire set of voxel timecourses, such as in independent component analysis (ICA, Figure 2). Below we describe each of these methods in more detail and provide a table (Supplementary Table 1) that lists which MS-related study employed which of these methods.

#### Correlation based analyses

FC as first introduced by Biswal et al. (27) made use of linear correlations [Pearson's correlation, but see (29, 30)]. More recent approaches employ partial correlations in order to remove the influence of, for example, physiological noise [e.g., (31)] or other confounding variables. Physiological and movement noise can systematically affect measured connectivity in multiple ways, despite having nothing to do with the process of interest (in this case, the communication between brain areas). If such confounding signals occurred uniformly all over the brain, then measures of statistical association, such as correlations, would increase, but uninformatively so. Regions at the edge of the brain, (i.e., mostly cortical regions), are more susceptible to movement-induced artifacts than regions in the middle of the brain, which can lead to biased reports of more severely reduced cortico-cortical connectivity than connectivity within the midbrain.

Furthermore, confounding signals that predominantly occur at certain locations (e.g., the lesion site) would lead to an attenuation of the statistical associations between the such locations and other brain areas, but it would not necessarily mean that the communication between these regions was affected. To our knowledge, neither such effects nor the impact of scanning parameters, such as voxel size on partial voluming effects (32) around lesions, have been investigated in the context of MS. However, they could pose particular problems in the case of MS, as patients may develop new lesions between repeated measurements.

##### Seed-to-voxel FC

Seed-to-voxel FC investigates FC between one so-called seed region with the rest of the brain (27) (Figure 1B), which yields one whole-brain map per seed region depicting how strongly each voxel is functionally connected to the chosen seed region(s) (Figure 1D). Such maps can demonstrate connectivity on the individual level, or they can be used for inferential statistics, for example when computing group differences in FC of a certain brain area. When selecting functionally defined seed-regions, one approach is to employ a localizer task. For example, for studying MS-related changes of the motor system, Lowe and colleagues used finger-tapping to identify motor-related regions-of-interest (ROIs) (33). Other studies have defined seed regions by using regions, identified via resting-state fMRI scans, that represent a certain network [such as the posterior cingulate cortex (PCC) (34) and ventromedial prefrontal cortex (vmPFC) (35) representing the entire default mode network (DMN) or a thalamic resting-state network (RSN) with right and left thalamus as seeds (36)].

To study changes of connectivity of dedicated functional systems, other researchers defined seed regions by using previously identified anatomical structures, such as the basal ganglia (37), the left caudate and the left thalamus (38), or the hand-knob (39). A lesion-based strategy selected seeds as regions that showed gray matter loss (40) or those cortical regions for which tractography suggested that their structural connectivity was affected by a white matter lesion (41).

##### Seed-to-seed FC vs. voxel-to-voxel FC

Both these approaches use pairwise (partial) correlations between BOLD timecourses. In a seed-to-seed analysis, one selects several brain areas, based on functional contrasts or existing atlases, and computes the correlations between the timecourses that represent the average signal per ROI (Figure 1E). Commonly used atlases include the anatomically defined Automated Anatomical Labeling Atlas (AAL) (42), a functionally-defined atlas recently proposed by Glasser et al. (43), or an atlas of resting-state networks created by Yeo et al. (44). This procedure yields a FC-matrix which can be used to, for example, study differences between groups (Figure 1G).

The disadvantages of averaging in the seed-to-seed approach are the loss of spatial resolution, thereby potentially overlooking meaningful results, either due to not taking all brain areas into account or by having lumped together functionally distinct voxels into one ROI. A spatially unconstrained alternative is to correlate all pairs of gray matter voxels without averaging, as done by Liu et al. (45). However, such a voxel-to-voxel approach results in huge correlation matrices, since commonly many tens of thousands of voxels are acquired, which, depending on the size of the dataset, may be a computational impossibility.

In contrast, seed-to-seed approaches substantially reduce the size of the data, as one only correlates timecourses from a few dozen seed regions, which can ease interpretation [e.g., (46), or (47), who only analyzed ROIs in the cerebellum]. Additionally, one increases the signal-to-noise ratio by averaging across voxels (since random noise contained in each voxel timecourse is attenuated or even canceled out by averaging over a large number of voxels). Although the majority of studies that investigated FC in MS chose seed-to-seed based approaches (see Supplementary Table 1), some groups used a version of the voxel-to-voxel approach to test whether any voxels shows high concordance with voxels in their neighborhoods. Wu et al. used this approach to investigate which regions lose their so-called regional homogeneity in MS (48).

#### Decomposition-based analysis

Another common strategy to identify brain networks is the decomposition of all voxel timecourses by means of *Independent Component Analysis (ICA)* into sets of spatially independent maps and their associated timecourses (49) (Figure 2). A single ICA component thus depicts groups of voxels that have a similar timecourse and are therefore considered to form a functional network.

Voxel values of a component map represent the contribution of the individual voxel to that component, which is typically normalized to a z-score. Thus, a change in a region's or voxel's z score indicates whether a voxel has increased or decreased its coherence with the rest of the voxels or regions that belong to the same functional network. Bonavita et al. (50) used a group-ICA (51) to investigate whether there is an overall difference in network strength between healthy controls (HC) and MS patients. Zhou et al. pushed such analyses even further by investigating whether the coherence of networks identified by ICA was affected by MS (52).

#### Applying graph theory to FC

Graph theory allows one to study networks formally. Brain-graphs consist of brain areas (nodes) and the connections between them (edges), which can be defined structurally or functionally. Graph theory provides a host of statistics that capture measures of functional segregation and integration. The starting point for calculating graph measures is usually a correlation matrix from a seed-to-seed FC analysis (26) (Figure 1G). For example, one graph-theoretic measure, the clustering coefficient, assesses the prevalence of clustered connectivity around individual nodes (a measure of segregation), which can be further used to describe at which level a network can be subdivided into clearly delineated and non-overlapping groups: so-called modules. On the other hand, path-length (the number of edges between nodes of interest) is a measure of functional integration and can be used to quantify the efficiency of a network (with efficiency being the inverse of path-length). A consistent finding of graph theory is that complex networks, be they the brain or social groups, follow a small-world organization (53), which is characterized by a few nodes that have many connections (hubs), whereas the vast majority of nodes only has a few short range connections. A small world organization keeps wiring cost low and efficiency high.

One consistent finding is that the overall efficiency is decreased in the MS-afflicted brain (54–56). Although the property of small-worldness is generally maintained in MS it appears to dissipate as the lesion load increases (57). Additionally, decreases in efficiency have been associated with more advanced clinical impairment (55, 57–61). For a more comprehensive review on graph theoretic measures in MS, see (62).

## Overview of selected literature

### Search strategy and selection criteria

References for this review were identified by searches of PubMed up until December 2017 and references from these articles that matched the search criteria but did not show up in the search. The search terms included “multiple sclerosis,” “fMRI,” “resting state,” and either “functional connectivity” or “graph theory.” There were no language restrictions. A complete description of the 86 matching articles is provided in Supplementary Table 1.

### Strategies for computing FC

There are three major strategies for analyzing FC data in the current MS literature. The first strategy involves comparing FC values from a group of MS patients to those from a group of HC. Commonly, the disease phenotype of the MS group is taken into account: typically RRMS (48, 57), SPMS (63, 64), or pediatric MS (65, 66). More specific disease characteristics, such as the lesion location, are rarely controlled for [for an exception see (41)].

The second strategy associates FC values of MS patients with clinical data, such as cognitive (50) and motor performance (67), fatigue (40, 68, 69), depression (70), or sleep disturbance (71). Here, either the respective symptom severity is used as a regressor of the FC data or the group of MS patients is subdivided into subgroups of preserved vs. impaired function.

The third strategy involves comparing the effect of treatment on changes of FC. Such studies typically involve placebo-controlled, pre- and post-measurements, and HC (72, 73).

### General differences between MS and HC

When comparing MS patients to HC, four general principles emerge that characterize FC within the MS brain: regions with high discriminative power, specific changes within functional resting-state networks, the breakdown of segregated modules, and a shift in hemispheric lateralization.

#### Regions with high discriminative power

Within the framework of FC, recent work has shown that MS predominantly affects *deep gray matter* regions (45, 46) resulting in both increased and decreased FC with other regions. The *thalamus* seems to play a more central role within the entire brain network in MS (74); specifically, the thalamus shows increased FC with the motor cortex (75, 76), the occipital and temporal cortices (45, 76, 77), the hippocampus and widespread subcortical areas and nuclei (45, 71), with the exception of the caudate (78), whereas it shows decreased FC with frontal regions (79). On the other hand, there is evidence that only some thalamic subregions exhibit decreased FC with the rest of the brain (80).

Although the *hippocampus* shows increased FC with the thalamus (71), there is recent evidence of increased FC between the hippocampus and Heschl's gyrus, the nucleus accumbens (71), and the amygdala (81). However, decreases of FC have been reported between the hippocampus and the cerebellum (70), the ACC, and the caudate nucleus (82, 83).

With respect to the *basal ganglia*, some nuclei, such as the caudate, show increased FC with frontal regions (48, 84), while other nuclei show decreased FC with fronto-parietal regions (85). In line with this, Cui et al. recently reported widespread differences of striatal FC between MS and HC (86). Lastly, the *amygdala* appears to increase its FC with the hippocampus and decrease its FC with certain nuceli of the basal ganglia, namely the putamen (81), and other widespread occipital and parietal regions (87).

Additionally, although MS predominantly seems to affect such deep gray matter regions, there is recent evidence that the FC of some cortical regions, such as the *medial temporal lobes*, is highly discriminative between MS patients and HC (45, 46).

#### Specific changes within resting-state networks

**Figure 3:**
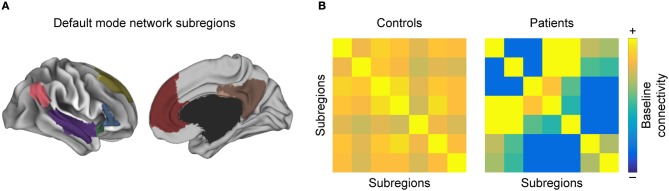
Intra-network functional connectivity (FC) changes in MS. Conceptual figure showing a simulated parcellation of the DMN, into **(A)** anatomical subregions. As discussed in the section “Regions with high discriminative power”, one often observes that **(B)** pairwise FC between those subregions is altered in MS with respect to healthy controls.

With respect to resting-state networks, there is a set of commonly reported networks that show altered FC properties in MS as compared to HC (46), chiefly the *visual network*, the *sensorimotor network (SMN)*, and the *default mode network (DMN)* (88, 89) (Figure 3).

##### Within-network coherence changes

When assessing the within-network FC coherence with ICA in MS compared to HC, many studies consistently report a breakdown in the visual network (45, 85, 90–92); although an investigation into early RRMS patients found no differences between groups (93) (to note, the vast majority of patients in these studies did not have optic neuritis). Similarly, ICA coherence decreases in the SMN (85, 90, 91). As for the DMN, although there is evidence for decreased ICA-network coherence therein (91), a number of other studies has failed to find such differences (85, 90), which is consistent with a comparable DMN network constitution between MS patients and HC, as assessed with measures of graph theory (59).

##### Within-Network Inter-Areal Connectivity Changes

When parceled into anatomical subunits, the DMN shows both increases and decreases in pairwise FC of these subunits when comparing MS patients to controls (52). In line with this observation, the PCC as a major hub of the DMN exhibited increased FC with other DMN subregions (33, 34, 50, 63, 94), while its FC with another DMN hub, the ACC, decreased (35, 50, 94). The ACC, in turn, exhibited increased FC with other DMN subregions (66, 95). Moreover, the temporal pole as part of the DMN showed increased FC to various other DMN subregions, which yields high discriminative power between MS patients and HC (45, 46). Finally, Rocca et al. (70) reported that the hippocampus decreases FC with many other DMN subregions, both cortical and subcortical. Additionally, regional homogeneity within DMN subregions appears to decrease, such as the insula and the caudate (48). Moreover, a recent study that analyzed five networks relevant to cognitive performance, including the DMN, found that the pairwise FC between the brain regions that make up the network is decreased in all networks in MS (96).

In the SMN, within-network connectivity changes as well in MS (36). The main drivers of the breakdown of coherence seem to be the premotor cortex (40, 64), the postcentral gyrus (63, 65, 97), and the thalamus (74–76). Regional homogeneity in the cerebellum, another part of the SMN, was found to decrease (98).

Finally, in the visual network, Gallo et al. (99) reported that the peristriate cortex exhibited decreased FC with other components. Also, the left and right primary visual cortex showed reduced coherence, while the anticorrelations of visual areas with the rest of the brain were lost in MS (100). In line with this, the efficiency within regions of the occipital cortex appeared to decrease (54, 101).

#### Breakdown of segregated modules

**Figure 4:**
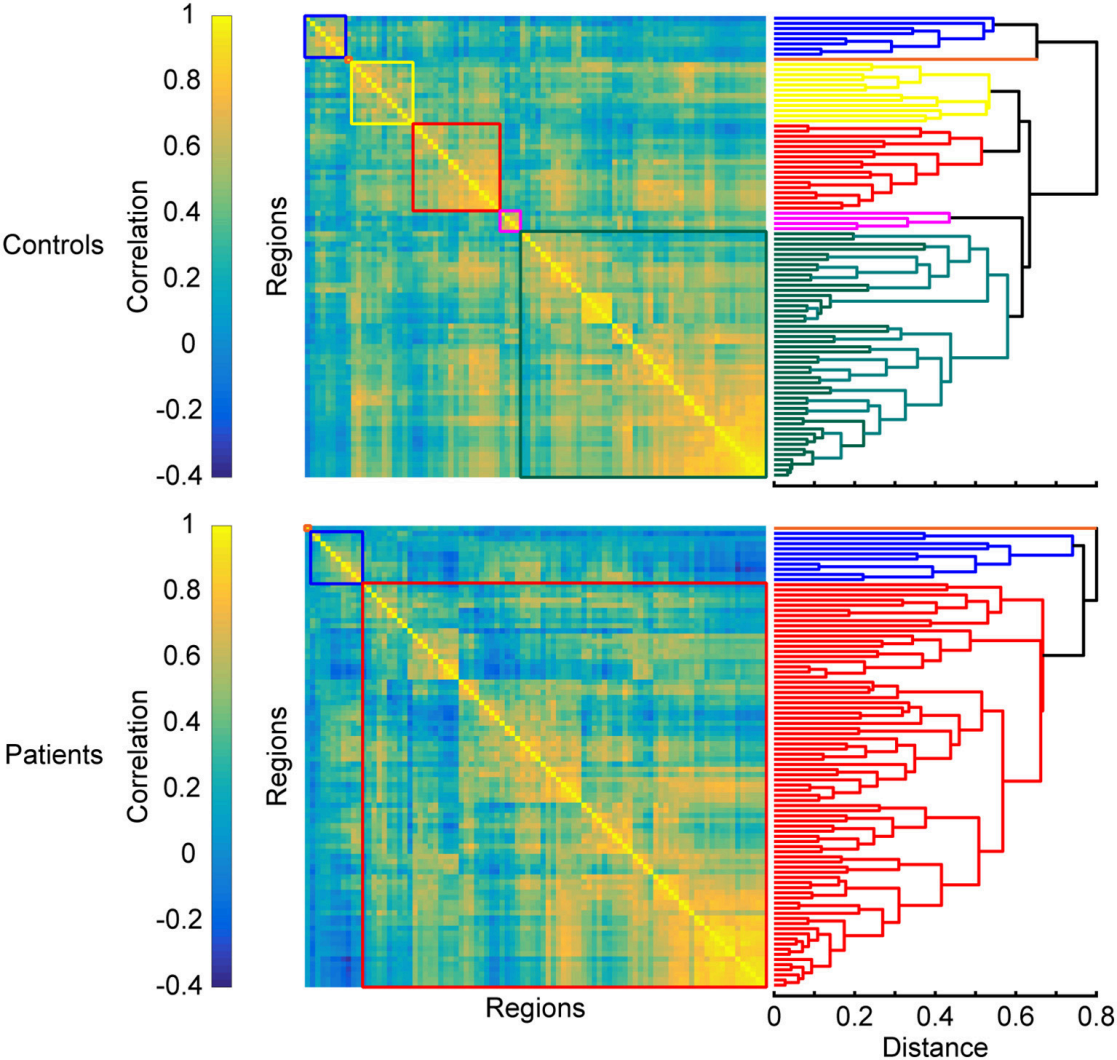
Changes in modular organization in MS. Top and bottom panels show matrices of pairwise interregional correlations and their underlying structure revealed by hierarchical clustering. Colored boxes correspond to the sub-clusters in the dendrograms on the right that are considered functional modules. A breakdown of functional modules automatically leads to an apparent formation of larger modules, which is often observed in patients when compared to healthy controls. Matrices are derived from simulated data and exaggerated for the purpose of illustrating the results discussed in the section “Breakdown of segregated modules”.

Another trend often observed in MS is the breakdown of segregated modules (Figure 4). Modularity is a graph-theoretic measure which describes the division of a network into functional units. The number of modules is reduced in MS compared to HC (59): posterior, central, and visual regions merge, while the cerebellum shows higher FC with hippocampal regions. The breakdown of modules increases with disease duration (102), which is also characterized by more hubs (55, 103) and can eventually lead to the formation of larger networks. Such larger networks can also be characterized by increased FC, which is found in MS between the thalamus and other deep subcortical nuclei within the SMN (75), the visual network and non-visual areas (100), the executive control and the salience network, and the working memory network and the attention network (91, 92) (see also section Formation of Larger Networks or Breakdown of Modularity? for further discussion).

#### Shift in hemispheric lateralization

A final recurrent observation is that FC decreases within the left cerebral hemisphere. This change has been reported between the left amygdala and occipitoparietal regions (87), the left insula and left precentral gyrus (48), and a more general isolation of the left hippocampus (82) and left amygdala (81, 83) from the rest of the brain. Conversely, FC within the right hemisphere reportedly increases, for example between the right caudate and the right dorsolateral prefrontal cortex (48) and between the right hippocampus and the right amygdala (46). Interestingly, this alteration in the hippocampus and amygdala opposes the pattern observed in their homolog areas in the opposite hemisphere. Similarly, the left frontoparietal network—but not the right—shows diminished coherence (104). Additionally, increased FC was found between the right working-memory network with the primary visual network in MS patients compared to controls, whereas the left working-memory network showed decreased FC with the SMN (91). Furthermore, an acute episode of optic neuritis was found to enhance FC emerging from the right cerebral hemisphere and the left cerebellar hemisphere (99), which would be expected given the opposite lateralization of the cerebellum. This general observation is further supported by a recent analysis that uncovered higher discriminative power of right-hemispheric regions when classifying between MS patients and HC: specifically increased FC between the right amygdala, the right hippocampus, and the right temporal pole (46).

**Figure 5:**
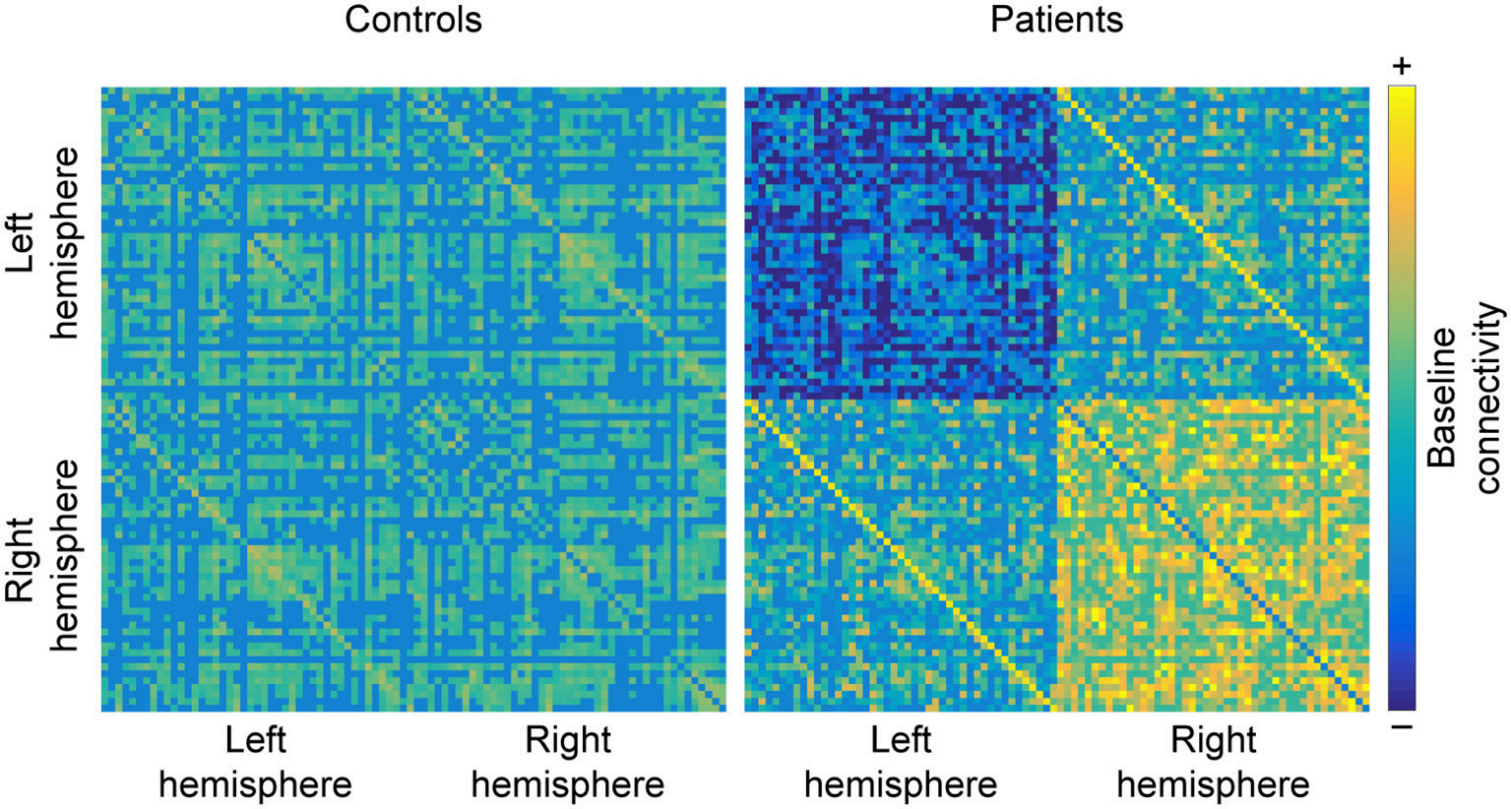
Hemispheric changes in MS. When compared to healthy controls, studying MS patients often reveals that functional connectivity (FC) decreases between regions of the left hemisphere and increases between regions of the right hemisphere. Additionally, homolog areas (depicted by minor diagonals) also show increased connectivity, which may be a byproduct of the loss of within-hemisphere communication. Matrices are derived from simulated data for the purpose of illustrating the results discussed in the section “Shift in hemispheric lateralization”.

Interestingly, homolog areas seem to show increased FC with each other in MS patients, reportedly found between the two mediotemporal lobes (52), the thalami (79), as well as the frontoparietal networks (85). However, there are also counterexamples, such as reduced FC between the left and right primary visual cortices in optic neuritis (100) and decreased FC between the hippocampi (70) (see Figure 5).

### What can FC tell us about MS symptom severity?

**Figure 6:**
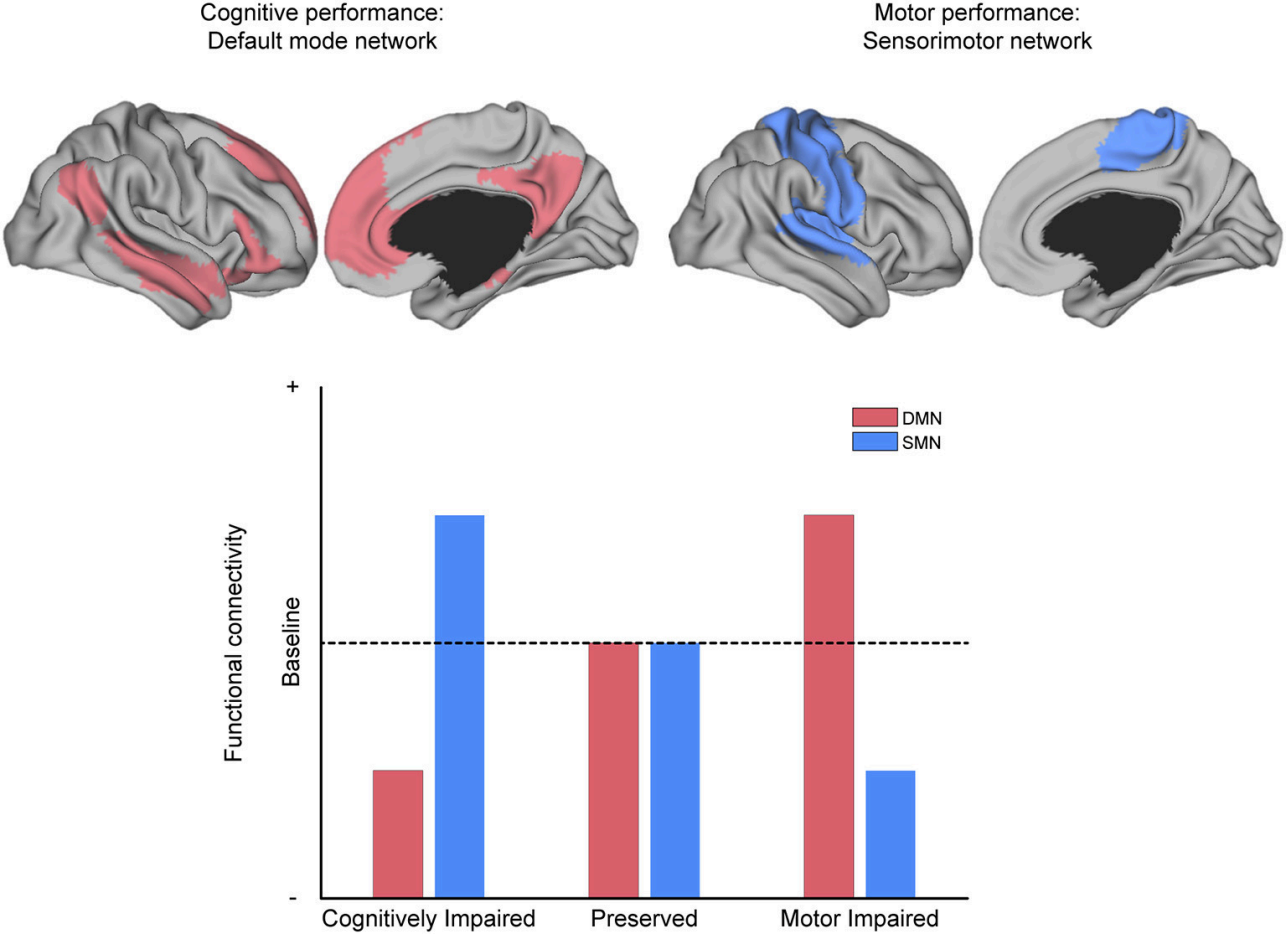
The relationship between MS symptoms and brain networks. Conceptual depiction of the often-reported match between the affected network and the specific symptom class from which patients suffer, as discussed in the section “What can functional connectivity (FC) tell us about symptom severity?” For example, the default-mode network (DMN, red) is associated with cognitive performance, and the sensorimotor network (SMN, blue) is associated with motor performance. MS patients with deficits in cognitive performance often have reduced FC within the DMN and increased FC within the SMN (as compared to patients without such deficits). The analog pattern of intra-domain decrease and extra-domain increase is observed for patients with deficits in motor performance, with respect to the SMN and DMN, respectively (as compared to patients without such deficits).

Apart from general differences in FC between MS and HC, prior investigations have revealed that cognitive and motor performance, as well as other neurological dysfunctions (e.g., fatigue, visual problems, depression, and sleep disturbance), are often associated with alterations in FC or change in network coherence. For example, stronger symptom severity has been linked to decreased FC in regions related to cognitive processing and increased FC in regions not typically associated with cognitive processing (Figure 6).

#### Task impairment in MS relates to decreased connectivity between and within task-specific regions

Generally, MS patients with impaired function often present with reduced FC in widespread brain regions as compared to patients with preserved function, who are often similar to the HC group [e.g., cognitive functions (90, 105), motor functions (67), sleep problems (71), and depression (70, 106, 107)]. Interestingly, such reductions in FC are often observed between brain regions that are classically linked to the function that is impaired in the MS group. For example, cognitive functions such as working memory or visual processing speed are typically associated with regions of the DMN; deficits attributed thereto in MS patients have been associated with reduced ICA-coherence of a major DMN-hub, the PCC (50, 108), which was even described as the most discriminant feature between cognitively impaired (CI) and cognitively preserved (CP) MS patients (109). Moreover, reduced FC between the cerebellum and other DMN-regions distinguished CI from CP pediatric MS (78). Additionally, decreased FC in the DMN was related to more severe memory impairment (110). Apart from the DMN, the working-memory network (92), attentional networks (60, 105, 109), and the executive-control network (60) have been associated with a decline in cognitive functions. To note, CP patients sometimes show even greater FC compared to HC in those networks (109).

Other brain regions that show positive correlations between cognitive performance and connectivity in MS are the medial prefrontal cortex and the frontal pole (35), the left insula (48), as well as the hippocampus (82, 111). However, increased FC of the right posterior hippocampus to the rest of the brain was elsewhere described as a main correlate of mnestic impairment (112).

Likewise in the motor system, decreased connectivity between domain-specific areas leads to impairment of domain-specific behavior. Motor dysfunction in MS has been associated with reduced FC in important SMN-hubs, for example from the sensorimotor/somatosensory cortices (39) and the cerebellum (84, 113) to the rest of the brain. Additionally, reduced motor performance co-occurred with reduced regional homogeneity in the cerebellum (98). Moreover, lower-limb spasticity could be improved with transcranial magnetic stimulation using intermittent theta burst stimulation, which increased the integrity (assessed with graph theory) of the contralateral primary motor cortex (114). Furthermore, motor-impaired MS patients have also been distinguished from motor-preserved MS patients based on reductions of FC in the thalamus (115) and in occipital regions (39, 67).

Lastly, visual problems in optic neuritis patients have been associated with reduced FC between visual regions (100) as well as reduced ICA-coherence in area V2 (99).

#### Task impairment in MS relates to an increase in connectivity between domain-external brain regions

In contrast to the findings discussed in the previous section, connectivity sometimes increases when comparing MS patients with an impaired function to those with preserved function (or even to the HC). For example, increased coherence in the right hippocampus/fusiform gyrus (45), increased coherence in several frontal networks (116), and increased coherence in the left motor network (64) have all been reported to correlate with more severe overall clinical disability. However, such measures are often given in EDSS or MSFC scores (i.e., multifunctional composite scores), which prevents accurate attribution of brain regions to distinct functions.

Interestingly, those investigations that analyzed a specific task and found increased connectivity in the impaired group revealed a pattern in which the brain regions with increased connectivity did not correspond to the brain regions classically associated with the task in question. For example, increased SMN-related connectivity (the network associated with motor function) was associated with *cognitive* deficits via increased FC between the thalamus and sensorimotor/occipital regions (117), between the thalamus and the cerebellum (36), or increased ICA-coherence in the thalamic RSN (105) and the medial visual network (85). Conversely, increased FC between *cognition*-related brain regions, such as between the left pallidum and the left anterior cingulate was instead associated with *motor* deficits (67).

### Training programs increase FC of domain-specific brain regions and networks

Recently, FC has been used as a part of the evaluation battery of therapeutic interventions in MS, and successful training programs have mostly been associated with increased FC in domain-specific brain regions and networks. For example, a cognitive computer training program reportedly increased FC between the PCC and bilateral inferior parietal cortices (106), important hubs of the DMN, which is relevant to cognitive performance. Moreover, a training program increased FC in the PCC, which was associated with better performance in the Stroop task (118). A successful cognitive training program conducted by Leavitt et al. (72) was related to higher FC between the left hippocampus and other cortical regions as well as between critical hubs of the DMN. In an earlier single-case study, the same group reported increased FC of the hippocampus to be associated with aerobic exercise as opposed to anaerobic exercise. This association also predicted enhanced cognitive performance (119).

Cognitively impaired MS patients who participated in cognitive rehabilitation programs reportedly showed: (a) increased ICA-coherence in cognition-related networks, which corresponded to improved neuropsychological performance, less severe depression, and better quality of life (120), (b) increased FC of the ACC and regions involving the non-dominant hemisphere, which corresponded to improved performance in the paced auditory serial addition test [PASAT, see (121)] (73), (c) increased FC between DMN-subnetworks, which corresponded to better performance in a working-memory task (122), and (d) altered thalamic FC: increased with the cingulum, precuneus, and bilateral parietal cortices, but decreased with the vermis and dorsolateral prefrontal cortex, which also corresponded to improved PASAT-performance (123).

## Discussion

### Recapitulation

MS is a progressive disease in which lesions may occur in any part of the CNS leading to a complex clinical manifestation of symptoms. These lesions directly affect either a region's function or the communication between brain areas and networks. Such changes in network communication and organization are the scope of FC studies, which may turn FC measures into biomarkers for studying MS.

In this review, we have reported a host of recent fMRI studies, which found that FC of MS patients is distinct from FC of HC. Specifically, FC between deep gray matter regions and the rest of the brain differs between MS and HC, most significantly for the thalami, hippocampi, basal ganglia, and amygdalae. Additionally, FC of a few cortical regions, such as the medial temporal lobes, tends to exhibit high discriminative power between MS and HC (see section Regions With High Discriminative Power). However, by taking a network perspective, several studies have demonstrated that resting-state networks in MS patients show reduced coherence (as measured with ICA and the inter-areal connectivity) (see section Specific Changes Within Resting-state Networks). Several other studies have shown that, throughout the disease course, the modular organization of networks gradually breaks down and larger networks form (see section Breakdown of Segregated Modules). Finally, a shift in lateralization seems to take place in MS patients, in that FC within the left hemisphere often decreases while tending to increase in the right hemisphere, with the exception that homolog areas often increase their mutual FC (see section Shift in Hemispheric Lateralization).

FC has been additionally associated with symptom-severity in MS yielding an interesting pattern: patients with impaired function often show reduced FC in domain-specific brain regions and networks (i.e., regions that are associated with the affected behavior) compared to patients with preserved function. However, there is also evidence that increased FC in domain-external brain regions and networks might be hampering performance (see section What can FC Tell us About MS Symptom Severity?). Lastly, successful training programs have almost always been associated with increases of FC in domain-specific brain regions and networks (see section Training Programs Increase FC of Domain-specific Brain Regions and Networks).

### Methodological observations and interpretational issues

When interpreting changes of FC in the MS brain, some statistical issues arise yielding caveats when interpreting general differences between MS and HC. As such, it is not yet clear whether the FC changes observed in MS are passive (i.e., merely natural consequences of the lesion occurrence) or active (i.e., the brain's attempt to minimize damage, referred to as neural plasticity). Moreover, whether such neural plasticity is adaptive (preventing impairment) or maladaptive (worsening impairment) is a topic of much discussion. When such issues are taken into account, however, we believe that FC can be investigated at an individual level and significantly improve the accuracy of MS differential diagnoses and prognoses.

#### Methodological observations

##### Highly discriminative regions: real effect or statistical artifact?

Firstly, we have seen that in MS FC of several brain regions, particularly deep gray matter regions, seems to be affected more often than other regions. When studying healthy brains, these regions are considered hubs, i.e., highly-connected regions through which large parts of the brain's overall communication is mediated (124).

The straightforward interpretation of this observation is that it is in the nature of MS to affect such hub regions. An observation which backs this hypothesis is that there are locations in the brain's white matter with an above-chance probability of having a lesion [e.g., the periventricular regions or the optic radiations, but see (8, 125)]. To the best of our knowledge, however, no study has investigated whether such lesion sites in MS preferentially connect to hub regions in gray matter. This observation could be clinically relevant given the purported link between a decrease in centrality of hub regions and increased severity of disability [see (126) for sensorimotor regions and (55) for the thalamus].

However, lesions in MS do also occur elsewhere in white matter, gray matter, and the spinal cord (127). Generally, in group-analyses of large data sets where the lesion locations are not controlled for, randomness alone would account for connectivity changes occurring more often in hub regions, given that these regions, by definition, have the most connections to other regions. Most investigations to date have studied MS groups without controlling for the lesion locations, thus providing little evidence for hub-specific effects resulting from MS. Such heterogeneity could also lead to seemingly contradictory results, in the sense that specific regions could show increased connectivity in one study but decreased connectivity in another. Additionally, gray matter atrophy, which is observed throughout MS disease progression (128), has been linked to FC changes in MS (83, 109) [but see (99) for opposing evidence], suggesting that further investigation into gray matter atrophy may be necessary for better understanding FC changes in MS.

One solution to this issue, and a step toward personalized medicine, would be to study single patients repeatedly. The advantage of repeated-measures studies in clinical neuroimaging of MS is that each patient serves as his/her own control (with respect to the number and location of the lesions), and repeated-measures designs allow one to study the timecourse of the disease. Recently, Droby et al. (41) employed such a personalized design in one patient by tracking the longitudinal effects of FC changes of an acute lesion, as it turned chronic, throughout a year. Having more highly-controlled and longitudinal records available will improve our understanding of how the individual disease progression corresponds to changes in FC and what role lesions play therein (129). However, FC is subject to substantial variability even within a single subject (130), which may reflect the normal variability of mental or physiological states (131). Whether such normal within-subject variability is low enough, compared to pathological changes of FC in MS, to allow for individualized FC-based diagnosis or prognosis is still an open question.

There are two main drawbacks to studying progressive diseases, such as MS, with repeated-measures designs: (a) lesions that were acute (active) during one measurement may be inactive in a following measurement but can reactivate throughout the disease course, even in the absence of clinical symptoms (132), and (b) the number of inactive lesions increases with time, rendering it difficult to attribute any changes in FC exclusively to the original lesion of interest. Nevertheless, we argue that, when studying such a heterogeneous disease, repeated measurements provide the greatest statistical power and highest achievable control over lesion location and load, thus offering the best possibility to improve differential diagnostic and prognostic information for the individual, which additionally counteracts the problem of small sample sizes.

##### Formation of larger networks or breakdown of modularity?

Another common observation is that FC networks appear to reorganize in MS. On one hand, these networks show reduced coherence (as measured with ICA and inter-areal connectivity), but on the other hand, their modular organization gradually breaks down, resulting in, what appears to be, larger networks. In our opinion this can be interpreted in at least two different ways: reduced coherence and formation of larger networks (a) are two different processes, or (b) they are one common statistical consequence of networks breaking down. As within-network coherence decreases, it is more likely that a statistical clustering procedure treats different regions as one group. Furthermore, less variability between different regions therefore can appear as increased FC. Rather than representing the formation of larger networks, this increase in FC between such networks can instead be explained by a decrease in variability and modularity.

#### Interpretational issues

##### What does increased or decreased FC mean?

It is important to note that there may not be a straightforward interpretation of increased or decreased connectivity between two brain regions. In our view, it is overly simplistic to interpret the change of FC between a pair of brain regions in isolation. For example, in the case of the breakdown of modularity we observe *both* a decrease in FC of nodes that belong to the same module *and* an increase in FC of nodes that belong to different modules (133, 134). Therefore, we strongly advocate a network-based approach when trying to interpret FC or when testing hypotheses related to changes of connectivity.

Another interpretational problem arises from the fact that the literature predominantly reports difference maps of changes in FC (regardless of whether the underlying FC exhibited a positive or a negative correlation), meaning that an increase in FC could be the result of reduced opponency (a previous inhibitory connection becoming weaker) or increased synchrony (a previous excitatory connection becoming stronger). While it thus seems advisable to report changes in negatively coupled regions separately from changes in positively coupled regions, this issue is complicated by the ongoing debate regarding how one should even properly compute FC. For example, one common approach involves removing physiological noise by subtracting out the global mean of all voxels. On the one hand, this method yields more stable estimates of FC, since information shared by all voxels is considered non-informative for differentiating the responses of different brain areas; on the other hand, this method has been criticized for inducing spurious negative correlations between brain areas, as the distribution of correlations is consequently “artificially” centered around zero [see (135) for a detailed treatise]. As such, depending on the strategy used for data preprocessing, the reader is faced with different interpretations of what, e.g., an increase in FC between two negatively correlated areas may indicate.

##### Are FC changes active (Compensatory Mechanism) or passive (Epiphenomenal) in MS?

Beyond such methodological issues, an additional question that remains open is that of the underlying mechanisms which actually drive FC changes in MS. Specifically, one could classify such mechanisms as either passive (a by-product of the lesion) or active (an attempt of the brain to maintain/restore function).

Functional changes may simply result from structural changes (136), which in turn can be passive or active. One approach to test the *passive* hypothesis would be to investigate the effects of virtual lesions in computationally modeled brain networks, where no in-built survival mechanisms should exist. Cabral et al. tested this idea and showed that structural damage changes the FC organization of a virtual brain network, which also manifested itself as decreased small-worldness of the network (137). This finding from a computational model corroborates the observation of decreasing small-worldness as a function of increasing lesion load in MS (57). More recently, Patel et al. used simulations to demonstrate that damage to structural connectivity can give rise to increased FC which they then compared to empirical FC within MS patients (133).

Despite the evidence for the passive hypothesis, there is strong evidence that a brain lesion triggers at least some degree of *active* recovery mechanisms. The potential of the brain to reorganize after injury, in order to minimize damage, is the well-known phenomenon of neural plasticity (a.k.a. neuroplasticity or brain plasticity) (138). This neuroplastic potential differs across people (139) (e.g., higher likelihood of younger people recovering function following brain damage than older people) and across brain regions (140) (e.g., higher likelihood of functions related to the frontal lobe recovering following damage than functions related to the post-central gyrus). Particularly, investigations into stroke patients have suggested that, if the left hemisphere is damaged, the right hemisphere may take over its functions (141). Interestingly, homolog areas appear to take over the functions of their injured counterpart, which was shown for both the sensorimotor cortices in stroke patients (142) and the inferior frontal gyri after experimentally induced aphasia using theta burst stimulation (143). Both these findings are consistent with results from MS studies: FC within the left hemisphere decreases, while it increases in the right hemisphere, and FC of homolog areas in the two hemispheres was shown to increase. These findings from MS studies corroborate those from stroke and aphasia studies, suggesting a general underlying mechanism for some form of active function recovery following neurological disorders.

##### Can FC/neuroplasticity be maladaptive in MS?

Apart from the active/passive distinction of characterizing FC changes, another characterization scheme addresses the perspective of whether neuroplastic changes aid a patient's recovery (i.e., they are adaptive) or rather contribute to the clinical impairment (i.e., they are maladaptive) (144). However, as we reviewed, increases in FC have been associated with both decreases and increases in performance on some tasks. This leads to two possibilities: either (a) increases in FC have no association to task performance or (b) the “adaptiveness” of FC increases might depend on where in the brain they occur and which functional domains are classically associated with that region. While FC increases within domain-specific brain regions might improve the patient's ability to perform the task at hand [e.g., cognitively preserved patients had higher FC between the DMN-hubs, MPFC, and PCC compared to cognitively impaired patients, see (109); and patients with preserved manual function had higher FC from sensorimotor regions to the rest of the brain, see (39)], it might simultaneously hamper the performance in domain-unrelated tasks [e.g., patients with upper limb motor disability had increased FC between the DMN-regions, left pallidum, and ACC compared to patients without such disability, see (67); and patients with cognitive deficits had increased FC between sensorimotor regions and the thalamus, see (117)].

This observation suggests that FC is neither purely adaptive nor purely maladaptive, but rather of combination of the two. It is adaptive in that domain-related task performance increases (following lesions in the pertinent gray/white matter), while the performance of domain-unrelated tasks can be seen as a maladaptive by-product, possibly owing to either a cost-benefit prioritization of the damaged function (for the sake of survival) or a more general decrease in flexible network communication.

### Improving the current standard in MS diagnostics with FC

Although the full scope of FC in MS is not yet understood, we believe that it can improve the differential diagnoses and prognoses of individual patients. Currently, the gold-standard of MS diagnoses are the McDonald criteria, revised in 2017, which provide five different manners of combining three criteria (i.e., the number of clinical attacks, the occurrence and locations of lesions, and CSF-specific oligoclonal bands) in order to make a definitive MS diagnosis (127). Often, a FLAIR T2 and a GD-enhanced T1-weighted MRI are sufficient to make such a diagnosis; however, these biomarkers do not seem to allow more fine-grained differential diagnoses (relating to symptom severity) and prognoses (18). In this regard, FC seems to be a promising biomarker in its ability to contribute additional, and more individualized, information. Different measures of FC already successfully predicted development of clinically definitive MS in a CIS patient group (45), discriminated between different subtypes of the MS disease spectrum (88), and distinguished patients with motor impairments from those without motor impairments (67).

The link between FC and symptom severity is especially striking. Often, decreased FC corresponds to increased disability, and the affected brain region tends to link to the type of symptom from which the patient suffers. Resting-state networks are task-associated, and thus it is important for future research to specify both the affected network and the type of clinical symptom that corresponded to FC changes. Currently, most research has provided correlations between FC and clinical data with composite scores such as the EDSS and MSFC, which comprise functions from many different domains (e.g., sensorimotor, cognitive, and visual), but in order to relate FC to symptoms in a biological manner, such functional domains that are affected by MS need to be more clearly distinguished.

Besides improving differential diagnoses, FC is also promising in its ability to predict the outcome of therapeutic intervention. One report from 2009 used task-based FC to monitor MS patients taking rivastigmine and domperidone (145). The authors demonstrated that patients who took a combination of both medications showed improved performance in a Stroop task when compared to those who took only rivastigmine; this improvement was associated with increased FC during task execution between homolog and other prefrontal and parietal regions. Additionally, that patient group reportedly showed reduced prefrontal-parietal FC within the dominant hemisphere during task execution. While this study made use of task-based FC, in the context of MS, no study so far has examined the effect of different pharmacological agents on FC in the resting state.

One drawback to investigating FC changes in a task-based framework (146, 147) is that particular tasks are known to recruit particular networks (148), thereby limiting what can be observed to the respective task-specific networks. A solution to this issue is to employ resting state-fMRI, which does not create such task-specific limitations and therefore allows one to investigate changes of network-level activity in the brain. This may be preferred to extracting resting-state FC from task-based FC (149), since one cannot guarantee a perfect model for task-effects that applies to all voxels. Instead, for example, Drysdale et al. (150) have recently demonstrated that, in a sample of more than 1,000 patients and controls, resting-state FC identified depression biotypes. Furthermore, only one of those subtypes benefited from transcranial magnetic stimulation therapy. Employing such an approach in MS could also reveal distinct biotypes that benefit more or less from different kinds of therapies or medications.

Lastly, with respect to longitudinal studies, Dogonowski and colleagues investigated how FC changes in MS during the weeks following a relapse (151). The authors reported that MS patients whose SMN coherence returned to a level similar to that of HCs after the relapse showed the best remission, as measured by reduced EDSS scores. This suggests that FC in MS is not static, and the longitudinal development of FC, as opposed to a “snapshot” FC measure, may better relate to the course of the MS disability. This notion is in line with a study by Faivre et al. (57) who showed that in MS patients with low disability rates at a baseline scan exhibited an increase of network efficiency at a two-year follow-up scan, whereas efficiency decreased in those patients who had high disability scores at baseline.

However, understanding the exact relationship between connectivity, the MS disease course, and disability severity will require further longitudinal investigations of FC in MS. Such an approach can help to identify specific patterns of FC that link to specific kinds of disease progression, thereby improving individual treatment. Similar approaches have already been successfully applied to other neurological disorders, such as Alzheimer's (152) and Parkinson's disease (153). Additionally, recent work has shown that FC may not be stationary (154), and therefore investigating FC fluctuations (130, 155) might provide additional information on the development of MS disease progression (87).

## Conclusion

This review aimed to synthesize recent research that employed the analysis of FC for the purpose of studying MS. We identified several common observations in MS, namely: (a) deep-gray matter regions show a higher likelihood of having altered FC (possibly due to these already being the most interconnected regions, also known as “network hubs”); (b) within-network coherence decreases, resulting in, what seems to be, the formation of larger networks, but can also be explained by a statistical consequence resulting from a decrease of modularity; (c) connectivity tends to decrease in the left hemisphere but increase in the right hemisphere; (d) patients with specific behavioral deficits show decreased connectivity within task-related networks but increased connectivity within task-unrelated networks. Such observations have surfaced because of the recent upsurge in applying connectivity-based analyses to multiple sclerosis. Crucially, combining connectivity analyses with longitudinal studies will likely provide the greatest potential for understanding adaptive and maladaptive changes in the MS brain on an individual level.

To reiterate, the concept of FC is based on the assumption that brain regions exhibiting a similar temporal evolution of activity share information and are thereby connected in a functional manner (27, 28). The literature of FC in MS that we have reviewed here has focused on statistical identification of disease-related changes in FC. Future work needs to address generative models of FC in MS, which reveal how certain connectivity changes can result from altered interactions in a coupled system (156, 157). However, MS presents a particularly difficult case for such an endeavor. One problem is that lesions and gray matter atrophy are not restricted to certain locations [despite some higher probability of lesions in periventricular regions (158)], which makes it difficult to pinpoint critical regions that generate typical patterns of altered connectivity. A second problem is that, by definition, MS-lesions occur at multiple sites, making it computationally difficult to estimate certain classes of generative models [such as dynamic causal modeling; (156)]. Therefore, at least for the near future, systemic measures such as breakdown of coherence or reorganization of network-hierarchies (159) may provide the most accessible means of investigating connectivity changes in MS. Furthermore, the combined use of FC and clinical scales as features in machine learning may yield different biotypes of MS that may respond differentially to different treatments, as recently demonstrated in FC-based biotyping in major depressive disorder (150).

## Author contributions

MT, RW, MWG and JS devised the review. MT conducted the literature review and provided a first draft. SL and JS extended the conceptual framework. MT, SL, and JS created the figures. RW provided insight on clinical aspects. All authors contributed to manuscript revision and approved the submitted version.

### Conflict of interest statement

RW is a Specialty Chief Editor for Frontiers in Neurology. The remaining authors declare that the research was conducted in the absence of any commercial or financial relationships that could be construed as a potential conflict of interest
