# Supplementary material for: Functional Connectivity in Multiple Sclerosis: Recent Findings and Future Directions
Source: Front Neurol. 2018 Oct 11;9:828. doi: 10.3389/fneur.2018.00828 (PMC6193088; doi:10.3389/fneur.2018.00828)
Supplement: Supplementary file 1 [file Table_1.DOCX]

Supplementary Material

**Functional Connectivity in Multiple Sclerosis: Recent Findings and Future Directions**

Marlene Tahedl, Seth M. Levine, Mark W. Greenlee, Robert Weissert, Jens V. Schwarzbach*

*Correspondence: [jens.schwarzbach@ukr.de](mailto:jens.schwarzbach@ukr.de)

| First author/year  Journal (Reference number in main article) | Objective | Subjects | Seed selection | FC method | Analysis strategy | Main findings |
| --- | --- | --- | --- | --- | --- | --- |
| Akbar et al., 2016  Plos One | Relationship between FC and SC changes in pediatric MS | 19 pediatric-onset MS vs. 16 HC | Whole-brain. | ICA | Group comparison of z-values of ICA components between MS and HC.  Correlation with SC measures. | - Pediatric MS: higher DMN component z-scores in the ACC and right precuneus; higher frontoparietal RSN component z-scores in the ACC and left middle frontal gyrus  - Higher ICA component z-scores in the right precuneus correlated with lower FA of the entire WM skeleton, especially of the CC and the right sagittal stratum |
| Akbar et al., 2016  Multiple Sclerosis Journal | FC changes in pediatric MS and its relationship to SC measures and clinical relevance | 16 cognitively intact pediatric MS vs. 15 HC | Whole-brain.  Atlas of cortical and subcortical regions: Cross-correlation of precuneus with all other brain regions. Seeds: 16 ROIs with highest correlation to precuneus. | STS (Pearson’s correlations) | Group comparison of FC matrices between MS and HC.  Correlation with neuropsychological data. | - Pediatric MS: higher FC of the precuneus with the ACC/frontal medial cortex/cerebellum; decreased FC of the thalamus with the right superior occipital region correlated with lower thalamic volume  - FC of left frontal medial cortex negatively correlated with cognitive performance, but only in the MS group |
| Basile et al., 2014  Multiple Sclerosis Journal | Integrity changes of the DMN and SMN in MS and relevance to MS-phenotypization | 34 RRMS vs. 14 SPMS vs. 22 HC | DMN and SMN: visually identified with whole-brain ICA | ICA | Group comparison of z-values of RSN networks between the MS groups and HC.  Correlation with neuropsychological data. | - MS: increased ICA component z-score in SMN and DMN; smaller differences between the RRMS and SPMS groups  - MS: increased ICA component z-score in the DMN correlated with worse cognitive performance  - SPMS, not RRMS: correlation of decreased SMN component z-scores in the precentral gyrus with increased motor disability |
| Bisecco et al., 2017  Multiple Sclerosis Journal | Contribution of FC changes in the SMN/DMN to fatigue in MS | 59 MS, classified as F (n=28) and NF (n=31) vs. 29 HC | DMN and SMN: visually identified with whole-brain ICA | ICA | Group comparison of t-values of ICA components between MS groups and HC.  Regression analysis to identify main predictors of fatigue. | - F vs. HC: increased DMN component z-score in the PCC; reduced in the ACC  - F vs. NF: increased DMN component z-score in the PCC; reduced FC in ACC; increased SMN component z-score in the primary motor/supplementary motor cortices  - DMN regions identified as main predictors of fatigue |
| Bonavita et al., 2011  Multiple Sclerosis Journal | FC alterations of the DMN in RRMS and contribution to cognitive performance | 18 cognitively impaired (CI) MS vs. 18 preserved (CP) RRMS vs. 18 HC | DMN: visually identified with whole-brain ICA | ICA | Comparison of z-values of ICA components between MS groups and HC. | Reduced DMN component z-score  in the ACC; reduced DMN component z-score in the core but stronger at the periphery of the PCC  🡪 more pronounced for CP |
| Bonavita et al., 2015  Journal of Neurology | RSN integrity changes following an 8-week computer-based cognitive rehabilitation program in MS and clinical relevance. | 32 cognitively impaired RRMS randomly assigned to computer cognitive (n=18) and no treatment (n=14) groups (baseline and follow-up) | DMN: visually identified with whole-brain ICA. | ICA | Group comparison of difference-maps of z-values of the DMN between the MS treatment groups.  Correlation with neuropsychological data. | Training group: increased ICA component z-score in the PCC and the bilateral inferior parietal cortex (IPC); negative correlation between ICA component z-score in PCC and Stroop performance (no such effects in the control group). |
| Bonavita et al., 2016  European Journal of Neurology | Contribution of RSN integrity to depression in MS | 16 depressed (D) MS vs. 17 non-depressed (ND) MS vs. 17 ND HC vs. 15 D-HC | DMN, salience and executive control RSNs: visually identified with whole-brain ICA | ICA | Group comparison of z-values of ICA components between the MS groups and the HC groups. | - D-MS vs. D-HC: decreased DMN component z-scores in the PCC  - D-MS vs. ND-MS: increased DMN component z-scores in the ACC, reduced in the PCC  - D-MS vs. D-HC/ND-MS: increased salience RSN component z-scores in the right supramarginal gyrus and middle frontal gyrus  - D-MS vs. ND-MS: increased ICA component integrity in the right inferior parietal cortex |
| Boutière et al., 2016  Multiple Sclerosis Journal | FC changes of the primary motor cortex after treatment with iTBS in spastic MS patients | 17 MS suffering from lower limb spasticity – randomly assigned to two groups receiving either real-iTBS or sham iTBS (baseline and 2 follow-ups) | Whole-brain. Cortical and subcortical atlas (116 ROIs) | STS (Pearson’s correlations of wavelet coefficients) | Group comparison of FC changes between the MS treatment groups over time.  Computation of graph theoretical measures (efficiency and laterality index).  Correlation with measures of spasticity. | - Real iTBS: FC laterality index changed at the expense of the stimulated area  - Spasticity improvement was correlated with changes of laterality |
| Chirumamilla et al., 2016  Conference Proceedings: IEEE | FC changes in RRMS with graph theory measures | 18 RRMS vs. 25 HC | Whole-brain.  Cortical and subcortical atlas (116 ROIs) | STS (Pearson’s correlations) | Computation of graph theoretical measures (clustering, global/local efficiency, modularity)  🡪 Group comparison of these measures between MS and HC. | - RRMS: increased modularity – increased FC with regions from the same module; decreased FC with regions from outside the module  - RRMS: increased efficiency in the insula, superior frontal gyrus, temporal pole |
| Cirillo et al., 2015  Multiple Sclerosis Journal | FC changes of the cerebellar dentate nuclei in pediatric MS and clinical relevance | 48 pediatric MS vs. 27 HC | Whole-brain.  Seeds: left/right cerebellar nuclei | STV (Pearson’s correlations) | Group comparison of FC maps between MS and HC.  Correlation with neuropsychological data. | - Pediatric MS: reduced FC between right dentate nuclei and bilateral caudate nuclei and left thalamus; increased FC between right dentate and left pre- and postcentral gyri  - CI: reduced FC between dentate nuclei and bilateral frontal, parietal and temporal regions; increased FC associated with better motor performance |
| Colasanti et al., 2016  Biological Psychiatry | Contribution of FC changes to depression in MS and relationship to with microglial activity | 11 MS vs. 22 HC | Whole-brain.  Seed: bilateral hippocampus | STV (Pearson’s correlations) | Group comparison of FC maps between MS and HC.  Correlation with depression scores (BDI).  Correlation with [^18^F]PBR111 uptake in PET imaging (reflecting microglial activity). | - Decreased hippocampal FC to subgenual cingulate and prefrontal parietal regions correlated with BDI scores and increased microglial activity  - BDI scores correlated positively with both in- and decreased hippocampal FC to widespread cortical/subcortical areas |
| Cruz-Gómez et al., 2016  Revista de la neurologia | Contribution of hippocampal FC and volume to long-term learning and information retention | 78 MS vs. 19 HC | Whole-brain.  Seeds: Left/right hippocampi | STV (Pearson’s correlations) | Group comparison of FC maps between MS and HC.  Correlation with neuropsychological data. | - MS: decreased FC between left hippocampus and brainstem/ cerebellum/fusiform gyrus/superior temporal gyrus  - MS: positive correlation between memory performance and hippocampal FC |
| Cruz-Gómez et al., 2014  Multiple Sclerosis Journal | RSN integrity changes in MS and relevance to cognitive performance | 60 MS, classified as CI (n=30) or 30 CP (n=30) vs. 18 HC | DMN, left/right frontoparietal and salience RSNs: visually identified with whole-brain ICA | ICA | Group comparison of t-values of RSNs between MS groups and HC.  Correlation with neuropsychological data. | CI vs. CP: decreased ICA component t-scores in all RSNs  CP vs. HC: decreased ICA component t-score in the left frontoparietal RSN |
| Cruz-Gómez et al., 2013  Plos One | Contribution of FC alterations to fatigue in MS | 60 RRMS, classified as fatigued (F, n=32) or non-fatigued (NF, n=28) vs. 18 HC | Regions of GM atrophy: identified with whole-brain VBM.  SMN: identified with whole-brain ICA. | STV (Pearson’s correlations)/  ICA | Group comparison of FC parameters between MS and HC.  Correlation with fatigue scores.  Comparison of z-values of SMN between MS groups and HC. | - RRMS: decreased FC between primary motor and somatosensory cortices  - F: decreased FC between SMA and associative somatosensory cortex  - NF vs. HC: higher SMN component z-score in the primary motor cortex  - NF vs. F: higher SMN component z-score in the premotor cortex |
| Cui et al., 2017  Frontiers in Neurology | Changes in striatal FC in MS, its reliability after 7 months, and clinical relevance | 20 RRMS (primary diagnosis) vs. 15 HC (baseline and follow-up after 7 months) | Whole-brain.  Seed: striatum, further subdivided into 6 ROIs on each hemisphere with an atlas | STV (Pearson’s correlations) | Group comparison of FC maps between MS and HC.  Correlation with EDSS scores. | RRMS: both increased and decreased FC between striatal components and in particular frontal/parietal regions correlated with worse EDSS |
| d’Ambrosio et al., 2017  Human Brain Mapping | FC alterations of SC- defined thalamic subregions in MS and clinical relevance | 187 MS, classified as CI (n=65) and CP (n=122) vs. 94 HC | Whole-brain.  Seed: Thalamus, further subdivided into 5 ROIs on each hemisphere based on FA values (own segmentation) | STV (Pearson’s correlations) | Group comparison of FC maps between the MS groups and HC.  Correlation with neuropsychological data. | - MS: increased FC for most thalamic subregions with widespread cortical and subcortical areas, specifically the left insula; reduced FC between motor/temporal thalamic subregions to deep gray matter and cerebellum  - CP vs. CI: Higher FC between thalamus and temporal areas  - Higher FC to caudate/cingulate cortex/insula correlated with better motor performance |
| Dogonowski et al., 2016  Journal of Neurology, Neurosurgery & Psychiatry | Integrity changes of the motor RSN following acute relapse and clinical relevance | 12 MS with acute relapse involving upper limb paresis (baseline: 24h within relapse and follow-up 6–21 days later) | SMN and two control RSNs: identified with whole-brain ICA and template-matching procedure | ICA | Comparison of ICA component z-score change between the different time points.  Correlation with EDSS scores. | Positive correlation between improvement in EDSS and reduction of SMN component z-scores in the bilateral mesial primary motor cortex |
| Dogonowski et al., 2013  Acta Neurologica Skandinavica | Integrity changes in motor regions in different MS-phenotypes and clinical relevance | 27 RRMS vs. 15 SPMS | Motor-RSN: identified with whole-brain ICA and template-matching procedure | ICA | Group comparison of z-values of motor-RSN between the MS groups.  Correlation with EDSS scores. | Motor-RSN component z-score in the left dPM positively correlated with clinical disability  🡪 strongest effect in the RRMS subgroup |
| Dogonowski et al., 2013  Multiple Sclerosis Journal | FC changes in the motor RSN in MS and contribution to motor performance | 42 MS vs. 30 HC | Motor-RSN: identified with whole-brain ICA | ICA | Group comparison of the spatial expansion of the motor-RSN between MS and HC. | MS: spatially enlarged motor-RSN component into deep subcortical nuclei (especially anterior and middle parts of the putamen, adjacent globus pallidus, anterior and posterior thalamus and subthalamic region) – not so at the cortical level. |
| Dogonowski et al, 2014  Neuroimage: Clinical | Regional homogeneity changes in MS and clinical relevance | 42 MS vs. 30 HC | Whole-brain. Cortical and subcortical atlas (90 ROIs).  Probabilistic cerebellar atlas. | VTV (Kendall’s coefficient of concordance) | Group comparison of whole-brain KCC maps between MS and HC.  Correlation with EDSS scores. | - MS: decreased KCC in upper left cerebellar hemisphere, with similar trends in the right cerebellar hemisphere  - MS: Negative correlation of FC at some posterior cerebellar regions and disability score. |
| Droby et al., 2015  Brain Imaging and Behavior | Longitudinal effects of acute and chronic MS lesions on FC | 5 MS with lesion at predilection site (MS+) vs. 4 MS without that lesion (MS–)/  1 MS developed an acute lesion at the defined ROI (baseline and 4 follow-ups) | Whole-brain.  Seeds: Cortical regions structurally connected to a common chronic lesion at the posterior periventricular region (identified with tractography) | STV (Pearson’s correlations) | Group comparison of FC maps across different time points, within and between MS+ and MS–.  Acute MS case: Tracking of FC changes across different time points (index reflecting average correlation of each ROI to all other brain voxels). | - MS+: increased FC in contralateral cuneus/precuneus and in the ipsilateral precuneus  - Acute MS: increased FC in parietal, frontal and cerebellar regions, which decreased at subsequent follow-ups. |
| Eijlers et al., 2017  Neurology | Contribution of changes in RSN hierarchy to cognitive impairment in MS | 332 MS, classified as CI (n=87), mildly CI (MCI, n=65) and CP (n=180) vs. 96 HC | Whole-brain. | VTV | Computation of graph theoretical measures for every voxel (centrality, degree).  Group comparison of these measures between the MS groups and HC. | - CI vs. CP/HC: increased centrality in regions compromising the DMN.  - MS vs. HC: decreased centrality in sensorimotor/occipital regions. |
| Eshaghi et al., 2015  Neuroimage: Clinical | Distinguish between MS groups with FC-/SC-based classifier | 30 NMO vs. 25 MS vs. 35 HC | DMN, SMN and visual network: identified with whole-brain ICA and template matching procedure. | ICA | Computation of a classifier to distinguish between MS phenotypes and HC, using ICA component z-scores, as well as SC measures (NAWM, GM volume, FA, and others). | - ICA component z-scores, next to NAWM, as most important predictor: 88% of cases correctly identified.  - MS: ICA component z-scores tended to be higher for all analyzed RSNs, but not significantly. |
| Faivre et al., 2012  Multiple Sclerosis Journal | FC-reorganization in MS and clinical relevance | 13 early RRMS vs. 14 MC | 8 RSN: visually identified with whole-brain ICA | ICA | Group comparison of z-values of each RSN between MS and HC.  Correlation with EDSS/MSFC scores. | MS: increased ICA component z-scores in 7/8 networks; negative correlation of MSFC scores and ICA component z-scores in the dorsal frontoparietal and prefronto-insular RSNs |
| Faivre et al., 2015  Journal of Clinical Neuroscience | Relationship of integrity of the hand RSN to activity during a hand task | 13 early RRMS vs. 14 HC | Dominant and non-dominant hand motor RSN: visually identified with whole-brain ICA | ICA | Computation of z-values of RSN components.  Correlation to motor activity during motor hand task. | MS: higher ICA component z-scores of the non-dominant hand RSN correlated with hyperconnectivity during task. |
| Faivre et al., 2016  Multiple Sclerosis Journal | Longitudinal FC changes in RRMS and contribution to disability progression | 38 RRMS, classified into 3 clinical subgroups according to MSFC scores, vs. 24 HC (baseline and 2-year follow-up) | Whole-brain. Cortical and subcortical atlas (90 ROIs) | STS (Pearson’s correlations of wavelet coefficients) | Computation of graph theoretical measures using FC matrices (nodal/local efficiency and degree)  🡪 Group comparison of these measures between the different MS groups | - MS vs. HC, baseline: higher long- and short-range nodal/local efficiency  - MS, follow-up: decrease of nodal/local efficiency) 🡪 comparable to HC  - MS with lowest disability at baseline: increase in all FC measures at follow-up  - MS with higher disability at baseline: decrease in all FC measures at follow up (here, FC decrease was correlated with disability progression) |
| Finke et al., 2014  Multiple Sclerosis Journal | Contribution of altered basal ganglia FC to fatigue in MS | 44 RRMS with different levels of fatigue vs. 20 HC | Whole-brain.  Seeds: left/right caudate nuclei, putamen, pallidum | STV (Pearson’s correlations) | Group comparison of FC maps between MS and HC for every seed region.  Correlation with fatigue scores. | - MS: decreased FC of basal ganglia to widespread cortical regions, no increases  - Fatigue severity negatively correlated with FC of basal ganglia nuclei with medial PFC, precuneus and PCC; positively correlated with FC between caudate nucleus and motor cortex |
| Gabilondo et al., 2015  Multiple Sclerosis Journal | Contribution of visual pathway damage and FC changes to performance of visual processing speed in MS | 30 MS without neurologic symptoms vs. 28 HC | Whole-brain.  Seed: medial-visual component | VTV (Pearson’s correlations) | Calculation of MVC’s “internal” FC (correlation of all individual MVC’s voxels’ compared to the correlation of all other brain voxels’ timeseries) and MVC’s “external” FC (mean correlation of all MVC’s voxels’ with all other brain voxel’s timeseries).  Group comparison of FC maps between MS and HC.  Correlation with visual processing speed. | - MS: lower internal FC of the medial-visual component  - MS: FC (both internal and external) of the MVC was stronger associated with task performance than in HC (both positive and negative correlation coefficients for different subparts of the MVC) |
| Gallo et al., 2012  Neurology | Visual RSN in RRMS with and without ON | 30 normal-sighted MS vs. 16 MS without optic neuritis (ON) vs. 14 MS with ON vs. 15 HC | Visual network: identified with whole-brain ICA | ICA | Group comparison of z-values of visual RSN between MS groups and HC.  Correlation with the amount of ON events. | - MS: Reduced V- RSN component z-score in the peristriate visual cortex  - ON: relatively stronger visual network component z-score in the extrastriate cortex and reduced in the right inferior peristriate cortex  🡪 positive correlation with the amount of ON events |
| Gamboa et al., 2014  Neuroimage | Topological properties of MS RSNs and contribution to cognitive performance | 16 early MS vs. 20 HC | Whole-brain. Cortical and subcortical atlas (116 ROIs) | STS (Pearson’s correlations) | Computation of a graph-theoretical measure using FC-matrices, reflecting whole-brain modularity.  Group comparison of this measure between MS and HC.  Correlation with neuropsychological data. | MS: increased network modularity and decreased functional integrity.  Modularity correlated negatively with PASAT performance |
| van Geest et al., 2016  Journal of Neurology | Contribution of FC changes to sleep disturbance in MS | 71 MS, classified as sleep-disturbed (n=23) vs. normal-sleeping (n=48) vs. 40 HC | Whole-brain. Seeds: left/right thalami and left/right hippocampi | STV (synchronization likelihood) | Group comparison of FC maps between MS groups and HC. | Sleep disturbed MS: decreased FC between thalamus and widespread parietal/frontal regions |
| De Giglio et al., 2016  Radiology | Thalamic-FC changes following an 8-week cognitive rehabilitation program in MS and contribution to cognitive performance | 24 CI MS, randomly assigned to cognitive-rehabilitation program (video-based) or wait-list group vs. 11 HC (baseline and follow-up) | Whole-brain.  Seed: Bilateral thalamus | STV (Pearson’s correlations) | Group comparison of FC maps between timepoints and between MS groups and HC.  Correlation with neuropsychological data. | - MS vs. HC, baseline: lower FC  - Training-MS, follow-up: increased FC with cingulum, precuneus and bilateral parietal cortex; decreased FC with vermis and DLPFC and better PASAT performance  - Wait-list MS, follow-up: increased FC involving cerebellum, right temporal lobe, frontal cortex. |
| Giorgio et al., 2015  The Journal of Neuroscience | RSN integrity and SC in RIS, as compared to RRMS and HC | 18 RIS vs. 20 HC and 20 RRMS | Several RSNs: visually identified with whole-brain PICA | ICA | Group comparison of z-values of RSNs between MS groups and HC.  Correlation of SC measures between the groups. | - RIS vs. HC: similar ICA network component z-scores  - MS vs. RIS/HC: increased ICA component z-scores in the SMN and WMN  - RIS vs. MS: better SC measures |
| González Torre et al., 2017  Multiple Sclerosis Journal | Contribution of FC changes to mnestic impairment in MS | 64 MS, classified as mnemonically impaired (MnI, n=33) vs. mnemonically preserved (MnP, n=31) vs. 18 HC | Whole-brain.  Seeds: Hippocampus, further subdivided into 7 ROIs on each hemisphere with an atlas | STV (Pearson’s correlations) | Group comparison of FC maps between the MS groups and HC. | - MnI>MnP>HC: FC between posterior hippocampus and several cortical areas  - Enhanced FC at the right posterior hippocampus as main correlate of mnestic impairment in the MnI group |
| Hidalgo de la Cruz et al., 2017:  Multiple Sclerosis Journal | Contribution of thalamic FC changes to fatigue in MS | 122 MS, classified as F (n=36) and NF (n=86) vs. 94 HC | Whole-brain.  Seeds: Thalamus, further subdivided into 5 ROIs on each hemisphere based on FA values | STV (Pearson’s correlations) | Group comparison of FC maps between MS groups and HC.  Correlation with different types of fatigue scores. | - F vs. NF/HC: altered thalamic FC to frontal/sensorimotor/cerebellar and insular regions  - Cognitive fatigue correlated to increased FC to precuneus and lower FC to cerebellum  - Physical fatigue correlated to increased FC with sensorimotor regions  - Psychosocial fatigue correlated to reduced FC with right insula |
| Hubacher et al., 2015  Restorative Neurology and Neuroscience | Long-term FC changes and task-fMRI changes following a cognitive rehabilitation program in juvenile MS and clinical relevance | 5 juvenile MS assessed to a 4-week cognitive training program (baseline, directly after training program and at 9-month follow-up) | Several RSN: identified with whole-brain ICA and template-matching procedure | STS (constrained maximally lagged correlations) | Comparison of individual changes in maximally-lagged correlations between networks and changes in task fMRI between the timepoints. Association with neuropsychological data. | - Directly after the training: Mixed results of in- and decreased CMLC across participants.  - Follow-up: increased CMLC between DMN-subnetworks, which was associated with better performance  - Patients without behavioral improvement showed no systematic FC changes |
| Janssen et al., 2013  Neuropsychologia | RSN integrity in MS and clinical relevance | 28 RRMS vs. 28 HC | 9 RSNs: identified with whole-brain ICA and template-matching procedure | ICA | Group comparison of z-values of each RSN between MS and HC. Correlation with disease severity composite. | - MS: reduced ICA component z-score in the motor and visual RSNs  - Stronger disease severity: reduced ICA component z-scores in the motor and executive control RSNs; increased medial-visual RSN component z-score to visuospatial regions |
| Jones et al., 2011  Archives of Neurology | FC alterations in the DMN following an acute lesion in the anterior thalamus | Case report of 1 acute onset amnesia case with an inflammatory lesion in the left anterior thalamus vs. 10 HC | DMN and other RSNs: visually identified with whole-brain ICA.  Extraction of 4 DMN-related ROIs using an atlas (left/right precunei and left/right PCCs) | STS (t-test) | Comparison of t-values of ROIs within the DMN between the individual and HC. | MS: Reduced DMN component z-score in the PCC on the left, lesion-ipsilateral, hemisphere |
| Koenig et al., 2013  American Journal of Neuroradiology | Sex-related FC differences in the DMN in MS | 16 male MS vs. 16 female MS vs. 16 male HC vs. 16 female HC | Whole-brain.  Seed: PCC, representing a major DMN-hub | STV (cross- correlations) | Group comparison of FC maps between the MS and HC groups. | - MS vs. HC: stronger FC from PCC to bilateral medial frontal gyri, the left ventral anterior cingulate, the right putamen and the left middle temporal gyrus  - Female MS: stronger FC between PCC and left dorsal lateral prefrontal cortex (dPFC) vs. female HC  - Male MS: stronger FC between PCC and left dPFC vs. female HC  - Male MS: weaker FC to caudate vs. female MS |
| Leavitt et al., 2013  Neurocase | Effects of aerobic exercise on FC in MS and contribution to cognitive performance | 1 MS exposed to aerobic exercise vs. 1 MS exposed to anaerobic exercise (both memory-impaired) (at baseline and at follow-up) | Whole-brain.  Seed: left hippocampus. | STV (Pearson’s correlations) | Comparison of FC difference-maps between the MS patient and the HC.  Correlation with neuropsychological data. | Aerobic exercise patient: Increased widespread FC of the left hippocampus, which correlated with better memory. |
| Leavitt et al., 2014  Journal of the International Neuropsychological Society | FC changes in the DMN in MS and contribution to memory performance | 43 MS with memory impairment (MI) and preserved memory (PM) | Whole-brain.  Seed: PCC, representing a major DMN hub | STV (Pearson’s correlations) | Group comparison of FC maps between the MS groups.  Correlation with neuropsychological data. | - MP vs. MI: increased DMN-FC  - DMN-FC correlated positively to memory function |
| Leavitt et al., 2014  Brain Imaging and Behavior | FC changes occurring with memory rehabilitation program in MS | 7 MS receiving cognitive training program vs. 7 placebo-controlled MS (baseline and follow-up) | Whole-brain.  Seeds: left/right hippocampi, representing hippocampal RSN; PCC, representing DMN | STV (Pearson’s correlations) | Group comparison of each FC-difference map between the MS treatment groups. Correlation with neuropsychological data. | Training group: increased FC between left hippocampus and cortical regions, and among critical DMN hubs along with behavioral improvements |
| Leonardi et al., 2013  Neuroimage | Patterns of FC dynamics in MS | 15 minimally disabled RRMS vs. 13 HC | Whole-brain. Cortical and subcortical atlas (88 ROIs). | STS (Pearson’s correlations) | Computation of dynamic FC fluctuation using FC matrices (“eigenconnectivities” of sliding time windows, calculated with PCA).  Group comparison of this index between MS and HC. | MS: widespread reduction of dynamic FC strengths (especially pronounced between the left amygdala and occipital/parietal regions, as well as between middle/posterior cingulate gyri and bilateral superior frontal gyri) |
| Liu et al., 2016  European Radiology | FC changes in CIS and differences of FC patterns between MS converters vs. non-converters at a 5-year follow-up | 20 CIS without conventional brain MRI lesions vs. 28 MS vs. 28 HC (baseline and 5-year follow up) | Whole-brain. | VTV | Group comparison of an index reflecting average connectivity of each voxel to rest of brain between the MS groups and across the timepoints. | - CIS vs. HC: decreased FC in visual areas, increased FC in temporal lobes  - MS vs. CIS/HC: decreased FC in left MFG and cuneus  - CIS-MS converters vs. non-converters: higher FC in right ACC and FG |
| Liu et al., 2015  Radiology | Thalamic FC/SC changes in MS and NMO and clinical relevance. | 37 MS vs. 39 NMO vs. 40 HC | Whole-brain.  Seed: Bilateral thalamus, further subdivided into 7 ROIs with an atlas | STV (cross-correlations/ weighted FC strength) | Group comparison of each FC/SC map between the MS groups and HC: intraregional FC with cross-correlation maps; interregional FC with weighted FC strength.  Correlation with neuropsychological data. | - MS vs. NMO/HC: decreased FC in several thalamic subregions  - FC changes did not correlate with any clinical markers, but some SC measures did |
| Liu et al., 2015  European Journal of Radiology | Thalamic FC in MS and clinical relevance | 35 MS vs. 35 HC | Whole-brain.  Seeds: left/right thalami | STV (Pearson’s correlations) | Group comparison of each of the FC maps between MS and HC.  Correlation with EDSS scores. | - MS: decreased FC between the thalamus and several brain regions including middle frontal and parahippocampal gyri, and the left IPL; increased intra- and interthalamic FC  - Negative correlation of inter-thalamic FC and disease duration |
| Liu et al., 2017:  Radiology | Topological organization of RSN in CIS and MS and clinical relevance | 34 MS vs. 34 CIS vs. 36 HC | Whole-brain. Cortical and subcortical atlas (90 ROIs) | STS (Pearson’s correlations) | Computation of graph theoretical measures (local/global efficiency, small-world measure) using FC matrices.  🡪 Group comparison of these measures between MS and HC. | - MS vs. HC: decreased global network efficiency, CIS with an intermediate value  - MS vs. HC: decreased nodal efficiency and FC in widespread cortical regions, involving occipital, temporal, frontal cortices and the insula  🡪 similar but weaker trend in CIS |
| Loitfelder et al., 2014  Brain Connectivity | Validation of a method to extract resting-state fMRI from block-design experiments | 12 MS vs. 18 HC | Whole-brain.  Seed: ACC. | STV (Pearson’s correlations) | Group comparison of FC maps between MS and HC.  Calculation of ACC-FC maps using task-fMRI data (block-design): Group comparison of the overlaps of FC-ACC maps calculated from task and resting-state between MS and HC. | Similar FC maps for MS and HC.  FC maps from task and resting state resulted in similar ACC-FC maps, both for MS and HC |
| Louapre et al., 2014  Human Brain Mapping | FC changes in MS and its relationship to SC abnormalities and cognitive performance | 15 CI RRMS vs. 20 CP RRMS vs. 20 HC | DMN, SMN, attention and visual RSNs: visually identified with whole-brain ICA | ICA | Group comparison of index reflecting functional integration of each network between the MS groups and HC (“functional integration”).  Correlation with SC measures (FA, AD, RD, GM atrophy). | - CP vs. CI: increased integration of attention RSNs  - CI vs. CP/HC: decreased integration of the DMN and attention RSNs  - Correlation between the integrity of the MPFC and the integrity of the PCC was predicted by PCC atrophy |
| Lowe et al., 2008  Human Brain Mapping | Relationship between SC and FC of motor cortices in MS | 11 MS vs. 10 HC | Whole-brain.  Seeds: right/left sensorimotor regions in M1 (peak active voxels in preliminary finger-tapping task) | STS (cross-correlations) | Group comparison of FC maps between MS and HC, reflecting correlations of all brain voxels to the seed regions.  Correlation with SC measures (FA, MD, TD, LD). | - MS: negative correlation between FC and TD  - MS/HC: positive correlation between FA and FC |
| Lowe et al., 2014  Brain Connectivity | Relationship of FC and SC changes in monosynaptically connected brain regions in MS | 19 RRMS vs. 16 HC | Whole-brain.  Seeds: right/left sensorimotor regions in M1 (peak active voxels in preliminary finger-tapping task)  more seeds: PCC, left/right hippocampus, SMC | STS (cross-correlations) | Group comparison of FC/SC values between MS and HC.  Cross-correlation with SC measures in three monosynaptic pathways associated with the Papez-circuit (FA, AD, RD, MD). | - MS and HC: positive correlation between FC and SC  - MS: reduced overall FC and SC |
| Lowe et al., 2002  Radiology | Coherence between task-active voxels and the same voxels during resting state in MS | 20 MS vs. 16 HC | Whole-brain.  Seeds: right/left precentral gyri (peak active voxels in preliminary finger-tapping task) | STS (cross-correlations) | Group comparison of FC maps during task and at rest between MS and HC. | MS vs. HC: Less coherence of the motor cortices during task vs. in the resting state. |
| Nejad-Davarani et al., 2016  Clinical Case Reports and Reviews | Contribution of FC changes to cognitive performance in MS | 10 RRMS vs. 20 HC | Whole-brain. Cortical and subcortical regions (94 ROIs). Identification of 5 RSNs, relevant for cognitive performance, from these regions from literature. | STS (Pearson’s correlations between all possible pairs of regions within each network) | Group comparison of FC changes between MS and HC. | MS: Reduced FC in each of the five analyzed networks |
| Parisi et al., 2014  Multiple Sclerosis Journal | FC changes following a 12-week rehabilitation program in MS and clinical relevance | 18 cognitive deficient MS, randomly assigned to cognitive treatment (n=9) vs. placebo treatment (n=9) (baseline and follow-up) | DMN, salience and executive control RSN: identified with whole-brain ICA | ICA | Group comparison of difference maps of z-values of RSN between the MS treatment groups and between.  Correlation with neuropsychological data. | - Increases in ICA component z-scores related to cognitive improvements, especially in cognitively relevant regions  - Changes in DMN component z-scores predicted cognitive performance and less severe depression  - ICA component changes in the executive control RSN predicted better quality of life |
| Parisi et al., 2014  Brain Imaging and Behavior | FC changes of the ACC following a cognitive rehabilitation program in MS and relevance to cognitive performance | 20 RRMS, randomly assigned to computer-cognitive training (n=10) vs. no-treatment group (n=10) (baseline and follow-up) | Whole-brain.  Seed: ACC, identified with an atlas | STV (Pearson’s correlations) | Group comparison of ACC-FC difference maps between the MS treatment groups.  Correlation with neuropsychological data. | - Treatment group, follow-up: increased FC of ACC with MFG and right IPL  - Control group, follow-up: decreased FC of ACC with right cerebellum and right ITG  - Training group: positive correlation between PASAT improvement and FC of ACC with right MFG |
| Petracca et al., 2017:  Scientific Reports | FC variability in PPMS and clinical relevance | 25 PPMS, classified as CI (n=13) and CP (n=12) vs. 12 HC | 3 RSNs: identified with whole-brain. STV: seeds placed in major hubs of dorsal attention, right attentional and executive control RSNs (from literature) | STV (Pearson’s correlations) | Calculation of a variability index for each ROI: standard deviation of the averaged time in each of predefined low frequency-bands.  Group comparison of the variability maps and the FC maps between MS groups and HC. | CI: increased variability and reduced synchronicity in the selected RSNs; increased FC between functionally distinct RSN |
| Petsas et al., 2015  Neurorehabilitation and Neural Repair | Integrity changes of the SMN and the cerebellar RSN following a simple motor task | 20 early RRMS vs. 14 HC | SMN and cerebellar RSN visually identified wither whole-brain ICA (before and after a 25-minute motor task) | ICA | Group comparison of z-values of RSN between the timepoints and between MS and HC. | - Before task, MS: higher ICA component z-scores in auditory and visual RSNs  - After task, MS: correlation of increased ICA component z-score in the cerebellar RSN and in the SMN  🡪 this intercorrelation was found specifically in MS after the task |
| Pinter et al., 2016:  Plos One | Reproducibility of longitudinal RSN-FC in MS | 20 stable MS vs. 14 HC (baseline and follow-up) | 9 RSNs: identified with whole-brain ICA and template-matching procedure; additionally some ROIs were identified in each network | STS  (intraclass correlation coefficient (ICC)) | Calculation of each network’s/ROI’s ICC changes between baseline and follow-up (reflecting the reproducibility of the network’s FC at the follow-up).  Group comparison of these maps between MS and HC. | Acceptable ICC for the ROI analysis, in both MS and HC  🡪 ICC for the RSN-analysis was not acceptable, neither in MS nor in HC |
| Prakash et al., 2011  Journal of the International Neuropsychological Society | Assessing FC differences between differing groups of PA in MS | 45 MS with different levels of physical activity (PA) | Whole-brain.  Seeds: left/right hippocampi, and others | STV (partial correlations) | Identification of regions with strongest FC to hippocampi.  Correlation of the correlation strength of those pairs with PA scores. | Increased FC between both hippocampi with the PCC correlated with higher PA. |
| Pravata et al., 2016  Multiple Sclerosis Journal | Contribution of dynamic RSN integrity changes following a cognitive task (PASAT) to cognitive fatigue in MS | 11 fatigued (F) MS vs. 11 non-fatigued (NF) MS vs. 12 HC (baseline and 2 follow ups: immediately after and 30 minutes after task) | Whole-brain.  Seeds: left caudate, left thalamus, SFG (all previously reported to be relevant in fatigue) | VTV (intrinsic connectivity contrast power (ICCP) | Group comparison of ROI-based FC changes between the MS groups and HC  Correlation with fatigue scores. | - F, immediately after task: increased ICCO between left SFG and left caudate nucleus  - F, 30 minutes after task: stronger ICC between left SFG and occipital, frontal and temporal areas, which increased over time after execution  - increased ICC between l-SFG hyperconnected and left anterior thalamus  🡪 both observations were correlated with perceived and assessed fatigue severity |
| Richiardi et al., 2012  Neuroimage | Discrimination between MS and HC using a whole-brain FC matrix | 22 minimally disabled MS vs. 14 HC | Whole-brain. Atlas of cortical and subcortical regions (90 ROIs) | STS (Pearson’s correlations) | Computation of classification algorithm using FC matrices between MS and HC | - MS: Widespread reduced pairwise FC; some increased FC in the right hemisphere  - Most discriminative regions: subcortical and temporal regions, more so on the contralateral side  - Classifier reached 82% sensitivity and 86% specificity |
| Rocca et al., 2014  Neurology | RSN integrity changes in pediatric MS and contribution to cognitive performance | 35 pediatric MS, classified as CI (n=16) and CP (n=19) vs. 16 HC | DMN: identified with whole-brain ICA and template-matching procedure | ICA | Group comparison of z-values of RSNs between MS groups and HC.  Correlation with neuropsychological data. | - CI vs. CP/HC: reduced ICA component z-score in the precuneus  - CP vs. CI/HC: increased ICA component z-score in the ACC  - ICA component z-score in the precuneus as predictor of cognitive impairment |
| Rocca et al., 2015  Human Brain Mapping | Changes of hippocampal FC and contribution to depression in MS | 69 MS vs. 42 HC | Whole-brain.  Seeds: right/left hippocampi | STV (Pearson’s correlations) | Group comparison of FC maps between MS and HC.  Correlation with depression scores. | - MS: decreased FC between hippocampi and several cortical-subcortical regions and the cerebellum  - MS: reduced FC correlated with increased depression |
| Rocca et al., 2014  Human Brain Mapping | Changes of RSN integrity and RSN-FC and clinical relevance | 44 pediatric MS, classified as CI vs. CP, vs. 27 HC | Several RSNs: identified with whole-brain ICA and template-matching procedure | ICA/  STS (constrained maximally lagged correlation) | Group comparison of z-values of RSNs between the MS groups and HC.  Group comparison of CMLC matrices between RSNs.  Correlation with neuropsychological data. | - Pediatric MS: reduced ICA component z-scores within various networks, which correlated with cognitive and motor performance (increased z-scores found only in parts of right attention RSN)  - Pediatric MS: decreased CMLC only between left WMN and DMN; increased CMLC between networks correlated with cognitive and motor performance  - CI vs. CP: decreased ICA component z-score in left WMN |
| Rocca et al., 2017:  Multiple Sclerosis Journal | FC changes in major RSNs in MS and clinical relevance | 215 MS vs. 98 HC | Whole-brain:  Seeds placed in major hubs of each of 7 previously defined RSNs . | STV (Pearson’s correlations) | For each network: Identification of spatial clusters with significantly different FC between MS and HC.  Group comparison of the z-scores of these clusters between MS and HC.  Correlation with neuropsychological data. | - MS: reduced ICA component z-scores in DMN/SMN, cognitive, cerebellar and thalamic RSNs; increased ICA component z-scores in visual/sensory and other subcortical RSNs - For most RSNs: reduced FC correlated with worse cognitive performance; except for the thalamic RSN, which showed the opposite pattern |
| Rocca et al., 2012  Neurology | Patterns of FC abnormalities in RRMS | 85 RRMS vs. 40 HC | Several RSNs: identified with whole-brain ICA and template-matching procedure | STS (constrained maximally lagged correlations)/  ICA | Group comparison of CMLC matrices between MS and HC.  Group comparison of z-values of RSN between MS and HC. | - RRMS: increased CMLC between executive control and salience network  - RRMS: decreased ICA component z-scores in regions of salience, executive control, working memory, DMN, SM and visual RSN and increased in regions of executive control and auditory RSN |
| Rocca et al., 2016:  Brain Structure and Function | Topological organization of FC in MS and clinical relevance | 256 MS vs. 55 HC | Whole-brain. Cortical and subcortical atlas (116 ROIs) | STS (Pearson’s correlations) | Computation of graph theoretical measures using FC matrices (small-world measures, clustering coefficient, path length, network degree, efficiency, assortativity, hierarchy)  🡪 Group comparison of these measures between MS and HC.  Association with MS subtypes and MS groups of differing cognitive impairment. | - MS: global network property abnormalities (lower network degree/global efficiency/hierarchy; higher path length/assortativity), loss of hubs, different lateralization (towards non-dominant hemisphere)  - Some graph-metrics could distinguish CP from CI, but not between the MS-subtypes |
| Romascano et al., 2015  Human Brain Mapping | FC/SC changes of the cerebellum during early MS and clinical relevance | 28 early RRMS vs. 16 HC | Cerebellum. Cerebellar atlas (30 ROIs) | STS (Pearson’s correlations) | Group comparison of combined FC/SC matrices between MS and HC.  Correlation with clinical data. | - Comparable cerebellar FC between early MS and HC.  - SC, but not FC measures, were correlated with behavioral performance. |
| Roosendaal et al., 2010  Neuroradiology | Hippocampal FC alterations in MS and relationship to hippocampal SC measures | 25 MS with intact spatial memory vs. 30 HC | Whole-brain.  Seeds: left/right hippocampi  (visually identified) | STV (Pearson’s correlations)/  STS (cross-correlations) | Group comparison of FC maps between MS and HC.  Correlation with hippocampal SC measures (GM volume, MD, lesion volume). | - MS, left hippocampus: decreased FC to anatomically connected areas (parietal, frontal, subcortical regions)  - MS, right hippocampus: decreased FC to some anatomically connected areas (left and right amygdala, left insular cortex)  - Decreased hippocampal FC correlated with more pronounced GM atrophy in the hippocampus |
| Roostaei et al., 2016  Neurology | Contribution of a variant in the sodium-channel encoding gene (SCN10A) to cerebellar FC changes in MS and clinical relevance | 161 relapsing onset MS vs. 94 HC, classified into two subgroups of the SCN10A polymorphism | Whole-brain.  Original seed: cerebellum. ROIs: identified as regions with significantly different cerebellar ICA z-scores between MS and HC | STS (Pearson’s correlations) | Group comparison of FC maps between MS and HC.  Correlation with MSFC/EDSS scores. | MS-carriers of the genotype (rs6795970AA) showed diminished FC between cerebellum with thalami and midbrain, which correlated with worse clinical status. |
| Sbardella et al., 2015  Multiple Sclerosis Journal | FC changes in MS and relationship with SC measures and clinical relevance | 30 RRMS vs. 24 HC | 11 RSNs: visually identified with whole-brain ICA | ICA/  STS (full and partial correlations) | Group comparison of z-values of RSNs between MS and HC  Group comparison of FC matrices between MS and HC.  Correlation with SC measures.  Correlation with neuropsychological data. | - MS: decreased ICA component z-scores in 5 RSNs (cerebellar, executive control, medial-visual, basal ganglia and SMN)  - MS: CC damage correlated positively with ICA component z-scores in the cerebellar and auditory RSNs  - ICA component z-score in medial visual network correlated negatively with processing speed  - MS: stronger inter-network correlations and anti-correlations |
| Sbardella et al., 2016  Multiple Sclerosis Journal | FC changes of the cerebellar dentate nucleus in MS and clinical relevance | 54 RRMS vs. 24 HC | Whole-brain.  Seeds: left/right dentate nuclei | STV (Pearson’s correlations) | Group comparison of FC maps between MS and HC.  Correlation with neuropsychological and clinical data, as well as with SC measures (FA). | MS: greater FC between dentate nuclei and cortical areas (esp. frontal and parietal regions)  🡪 within these regions: inverse correlation between FC and clinical impairment  - Higher FC correlated with higher FA |
| Schoonheim et al., 2013  Multiple Sclerosis Journal | Network integrity in MS and clinical relevance | 128 MS vs. 50 HC | Whole-brain.  Seeds: Regions of altered centrality, identified with whole-brain eigenvector centrality mapping (ECM). | VTV (ECM)/  STV (Pearson’s correlations) | Comparison of ECM-maps between MS and HC (reflecting the centrality of each voxel).  Group comparison of FC-maps between MS and HC.  Correlation with EDSS scores. | - MS: increased EC in the bilateral thalamus and PCC; decreased in sensorimotor and ventral stream areas  - MS: SM-EC decrease correlated to higher EDSS, ventral stream-EC decrease correlated to worse cognition |
| Schoonheim et al., 2015 Neurology | Thalamic FC/SC in MS and relevance to cognitive performance | 157 MS, classified as CP, mildly CI (MCI) and severely CI (SCI) vs. 47 HC | Whole-brain. Cortical and subcortical atlas (92 ROIs).  Seed: Bilateral thalamus | STS (synchronization likelihood) | Group comparison of SL matrices between the MS groups and HC.  Correlation with SC measures. | - Severely CI vs. mildly CI/CP/HC: increased thalamic FC with widespread cortical, especially sensorimotor, frontal and occipital regions  - FC measures did not correlate with SC measures |
| Schoonheim et al., 2012  Multiple Sclerosis Journal | Gender-related FC differences in male and female MS vs. matched HC and contribution to cognitive performance | 15 male MS vs. 15 female MS vs. 15 male HC vs. 15 female HC | Whole-brain. Atlas of cortical and subcortical regions (116 ROIs) | STS (synchronization likelihood) | Group comparison of SL matrices between MS and HC.  Computation of efficiency measures using SL-matrices.  Correlation to neuropsychological data. | Male MS: Widespread decreased SL and network efficiency, which correlated to reduced visuospatial memory. |
| Shu et al., 2016  Scientific Reports | Changes of FC and SC in CIS and MS and clinical relevance | 41 CIS vs. 32 MS vs. 35 HC | Whole-brain.  Cortical and subcortical atlas (90 ROIs) | STS (Pearson’s correlations) | Computation of graph theoretical measures using FC matrices (network strength, global/local efficiency, path length, clustering, small-world parameters)  🡪 Group comparison of these measures between the MS groups and HC.  Correlation with SC matrices.  Correlation with neuropsychological data. | - CIS vs. HC: no difference in FC; only in SC  - MS vs. HC: decreased local efficiency and clustering coefficient  - Changes in FC and SC measures were correlated only in the SMN and visual areas.  - FC measures were not correlated with neuropsychological measures (some SC measures were correlated) |
| Tewarie et al., 2015  Human Brain Mapping | Contribution of thalamic FC alterations to clinical status in MS | 86 MS vs. 21 HC | Whole brain.  Cortical atlas (78 ROIs).  Seeds: left/right bilateral thalami | STS (Pearson’s correlations) | Computation of graph theoretical measures (connectivity, clustering, path length, minimum spanning tree).  🡪 Group comparison of FC maps and graph measures between MS and HC.  Comparison with analog measures derived from MEG.  Correlation with clinical data. | - MS: increased thalamic FC to occipital and temporal regions, which was associated with worse clinical scores.  - Graph measures: no differences between MS and HC.  - MEG graph measures as better predictors of clinical scores than fMRI graph measures. |
| Tona et al., 2014  Radiology | Thalamocortical FC changes in MS and contribution to cognitive performance | 48 RRMS vs. 24 HC | Whole-brain.  Seeds: left/right thalami, representing the thalamic RSN | STV (Pearson’s correlations) | Group comparison of FC maps between MS and HC.  Correlation with neuropsychological data. | - MS: increased FC for the cerebellum, basal ganglia, hippocampus, cingulum, temporo-occipital, insular, frontal/parietal cortices; decreased FC for thalamus, cerebellum, cingulum, insular/prefrontal/parieto-occipital cortices  - Correlation of higher thalamic, cerebellar and some cortical/cerebellar lobule FCs and worse PASAT scores |
| Wojtowicz et al., 2014  Multiple Sclerosis Journal | FC changes in the DMN in MS and contribution to performance variability in processing speed | 18 female RRMS vs. 16 female HC | Whole-brain.  Seeds: PCC and vmPFC, representing the DMN | STV (Pearson’s correlations) | Group comparison of FC maps between MS and HC.  Correlation with neuropsychological data. | MS: more stable task performance correlated with greater FC between ventral medial prefrontal cortex and the frontal pole |
| Wu et al., 2015  Investigative Ophthalmology and Visual Science | Effects of acute ON on FC and contribution to visual performance | 15 CIS with acute ON (50 days after onset) vs. 18 HC | Whole-brain.  Seed: left primary visual cortex | STV (Pearson’s correlations; phase lag index) | Group comparison of FC maps between the timepoints and between ON and HC.  Correlation with scores on visual acuity. | - ON: reduced FC within the visual system, loss of homotopic FC between left and right V1s, loss of anticorrelations between visual system and nonvisual network  - Stronger FC between visual regions correlated with better visual acuity scores |
| Wu et al., 2016  Neuropsychiatric Disease and Treatment | Regional homogeneity changes in RRMS and clinical relevance | 22 RRMS vs. 22 HC | Whole-brain.  Seeds: Regions of different synchronization, identified with previous regional homogeneity analysis | VTV (KCC)/  STV (Pearson’s correlations) | Group comparison of KCC maps between MS and HC.  Group comparison of FC maps between MS and HC.  Correlation with neuropsychological data. | - MS: decreased KCC in left insula, associated with worse PASAT  - KCC in the right caudate negatively correlated with disease duration  - MS: decreased FC between left insula and left precentral gyrus; increased FC between right caudate and right dorsolateral prefrontal cortex 🡪 no clinical correlations found for FC maps |
| Zhong et al., 2016  Brain Imaging and Behavior | Classifier to distinguish motor preserved and impaired MS and HC (FC and/or SC-based) | 26 MoP MS vs. 25 MoI MS vs. 21 HC | Whole-brain. Cortical and subcortical atlas (86 ROIs) | STS (Pearson’s correlations) | Computation of SVM (leave-one-out) to distinguish the MS groups and HC using FC matrices. Separate SVMs were computed for FC and SC data, as well as one SVM for the combined data.  Chi-square test to assess distribution of correct and incorrect classifications. | FC-only SVM was sufficient for correct classification of upper limb motor disability (SVM considering both FC and SC metrics reach accuracy of >85%) |
| Zhong et al., 2016  Human Brain Mapping | Changes of SMN-FC in MS and contribution to hand-motor performance | 26 MoI MS vs. 17 MoP MS vs. 20 HC | Whole-brain.  Seed: Left hand-motor knob, identified from literature | STV (cross-correlations) | Group comparison of FC maps between MS groups and HC.  Correlation with neuropsychological data. | - MoP vs. MoI/HC: stronger FC in structurally intact visual information processing regions  - MoI vs. MoP/HC: weaker FC of sensorimotor and somatosensory association cortices; more structural damage  - Both MS groups: LM1-FC correlated with faster 9HPT (not in HC) |
| Zhou et al., 2016  Frontiers in Human Neuroscience | Thalamocortical integrity (SC and FC) to gain insight into MS-related functional plasticity | 20 minimally disabled RRMS vs. HC | Cortical regions only.  Cortical atlas (86 ROIs).  Seed: Bilateral thalamus, further subdivided into 7 ROIs from literature. | STV (Pearson’s correlations) | Group comparison of FC maps between MS and HC.  Correlation to SC measures. | - MS: increased FC in right temporal pole, which correlated positively with lesion load  - MS: increased FC to regions within primary motor cortex, occipital cortex, PFC and temporal cortex; decreased FC to PFC-region |
| Zhou et al., 2014  Plos One | Relationship between SC and FC changes within the DMN in MS and clinical relevance | 24 minimally disabled RRMS vs. 24 HC | DMN: identified with whole-brain ICA and template-matching procedure.  Parcellation of DMN into 6 subregions. | STS (Pearson’s correlations) | For subregions group comparison of FC and SC parameters (FA, MD, AD). Correlation of FC and SC indices.  Correlation with EDSS scores. | - MS: decreased SC and increased FC of paired DMN subregions  - Both FC and SC values were correlated to EDSS, with SC being stronger predictors of EDSS scores |
| Zhou et al., 2015  Neuropsychiatric Disease and Treatment | FC of amygdala and hippocampus in MS and relationship to SC measures | 23 minimally disabled RRMS vs. 23 HC | Whole-brain.  Seeds: left/right amygdalae; left/right hippocampi | STV (Pearson’s correlations) | Group comparison of each FC map between MS and HC.  Correlation with SC measures. | - MS: Reduced FC to bilateral putamen; increased FC between bilateral hippocampus and left amygdala  - Increased FC correlated with WM tract damage to the right hippocampus and right amygdala |

**Supplementary Table 1**. Overview of studies that investigated functional connectivity in the resting state in multiple sclerosis (see section “Search strategy and selection criteria” for further details on the selection process). Abbreviations (in alphabetical order): ACC: anterior cingulate cortex; BDI: Beck’s Depression Inventory; CI: cognitively impaired; CIS: Clinically Isolated Syndrome; CMLC: constrained maximally-lagged correlations; CP: cognitively preserved; D: depressed; DMN: Default Mode Network; dPM: dorsal premotor cortex; ECM: eigenvector connectivity mapping; EDSS: Expanded Disability Status Scale; F: fatigued; FA: Fractional anisotropy; FC: Functional connectivity; FG: fusiform gyrus; GM: gray matter, HC: healthy control; ICA: independent component analysis; ICC: intraclass correlation coefficient; ICCP: intrinsic connectivity contrast power; IPL: inferior-parietal lobe; ITG: inferior temporal gyrus; KCC: Kendall’s Coefficient of Concordance; MFG: medial frontal gyrus; MI: memory impaired; MnI: mnemonically impaired, MnP: mnemonically preserved; MoI: motor impaired; MoP: motor preserved; MP: memory preserved; MPFC: medial prefrontal cortex; MS: Multiple Sclerosis; MSFC: Multiple Sclerosis Functional Composite; MVC: medial-visual component; n: sample size; ND: non-depressed; NF: non-fatigued; ON: Optic Neuritis; PA: Physical activity; PCA: principal component analysis; PCC: posterior cingulate cortex; PET: positron emission tomography; PICA: probabilistic independent component analysis; (d)PFC: (dorsal) prefrontal cortex; RIS: Radiologically Isolated Syndrome; ROI: region of interest; RR: relapsing-remitting; RSN: resting-state network; SC: structural connectivity; SFG: superior frontal gyrus; SL: synchronization likelihood; SMA: supplementary motor area; SMN: sensorimotor network; SVM: support vector machine; TD: transverse diffusivity; VBM: voxel-based morphometry; vmPFC: ventromedial prefrontal cortex; WMN: working memory network; vs.: versus
